# System-level design trade-offs in magnetic hyperthermia RF instrumentation: a power electronics and electromagnetic design review

**DOI:** 10.1186/s12938-026-01633-3

**Published:** 2026-10-07

**Authors:** Serhat Ilgaz Yöner, Alpay Özcan

**Affiliations:** 1 Department of Clinical Science, Intervention and Technology (CLINTEC), Karolinska Institutet, Alfred Nobels allé 8, Huddinge, 141 52 Sweden; 2 Institute of Biomedical Engineering, Boğaziçi University, Kandilli Campus, Istanbul, 34684 Turkey; 3 Electrical and Electronics Engineering Department, Boğaziçi University, İstanbul, 34342 Turkey; 4 Magnetic Medical Devices Laboratory, Boğaziçi University, İstanbul, 34342 Turkey; 5 Systems Science and Mathematics Laboratory, Boğaziçi University, İstanbul, 34342 Turkey; 6 Center for Targeted Therapy Technologies (CT3), Boğaziçi University, İstanbul, 34684 Turkey

**Keywords:** Magnetic hyperthermia, Instrumentation, Magnetic fields, Induction heating

## Abstract

****Purpose**:**

Magnetic hyperthermia (MH) is a promising localized cancer therapy based on magnetic nanoparticle (MNP)-mediated heating. Although research has extensively focused on MNP development, therapeutic efficacy and safety also depend critically on the engineering of the induction system. This review aims to provide an engineering-focused analysis of contemporary MH instrumentation and its design trade-offs.

****Methods**:**

The instrumentation architecture is examined through a dual-domain evaluation of the power electronic and electromagnetic design chain. RF generator architectures are analyzed across the DC supply, oscillator, amplifier, and impedance matching stages. RF coil designs are evaluated according to geometry, inductance, and cooling strategies. An ordinal scoring framework is used to compare representative implementations and assess the influence of architectural choices on energy delivery and thermal stability, providing a structured basis for relative comparison rather than a definitive performance ranking.

****Results**:**

The analysis demonstrates that MNP performance is fundamentally constrained by the magnetic field generated by the complete instrumentation chain. Power-stage stability, impedance matching, coil characteristics, cooling, and spatial field homogeneity collectively determine the achievable operating conditions.

****Conclusion**:**

Reliable MH requires system-level optimization rather than isolated MNP development. By clarifying key hardware trade-offs and their effects on field generation, this review supports the design of more standardized and reproducible MH induction systems.

## Introduction

MH has emerged as a compelling modality in the landscape of oncological therapeutics, leveraging the heat-dissipating properties of MNPs when subjected to low-frequency alternating magnetic fields (AMF) [1]. The foundational concept of using inductively heated particles for localized tissue heating was first demonstrated by Gilchrist et al. [2], who applied selective inductive heating to lymph nodes using magnetic particles nearly seven decades ago. The core premise of MH lies in its ability to induce localized hyperthermia, typically raising intratumoral temperatures to the $42{-}45^{\circ }\mathrm{C}$ range to trigger apoptosis or enhance the efficacy of co-administered treatments [3, 4]. This method is particularly promising due to its non-invasive nature and high spatial specificity; by functionalizing MNPs to target specific bio-markers, heat can be delivered directly to malignant cells with minimal thermal impact on surrounding healthy tissues [5, 6].

As a therapeutic tool, MH provides a critical synergy with existing treatment standards. It serves as a potent sensitizer for radiotherapy and chemotherapy by increasing tumor oxygenation and blood flow, effectively addressing the limitations of the hypoxic tumor micro-environment [7, 8]. Furthermore, for deep-seated or inoperable lesions where surgical resection is high-risk, MH presents a repeatable, non-ionizing alternative [9, 10]. Unlike traditional radiation, which is limited by cumulative dose toxicity, the magnetic fields employed in MH allow for multiple treatment cycles, broadening the scope of long-term patient management [11].

Current literature reveals a profound concentration of research effort on the material science domain, specifically the optimization of MNP morphology and surface chemistry to enhance the Specific Loss Power (SLP) [12, 13]. However, the translation of these material advancements into consistent clinical outcomes is fundamentally dependent on the instrumentation that facilitates the energy transfer [5, 14]. At present, the electronic circuitry, comprising the RF generator and coil exhibits a significant lack of standardization. Many experimental platforms rely on heterogeneous, often unoptimized hardware architectures that lack the precision in amplitude–frequency control and field homogeneity necessary for rigorous clinical reproducibility [15–19].

This hardware-centric challenge is rooted in the fact that MH therapy is defined by a series of cascaded events. In this technological hierarchy, the RF generator serves as the foundational stage, providing the power and frequency stability required to drive the RF coil. The coil, in turn, translates this electrical energy into the precisely controlled magnetic field that excites the MNPs. The final cancer therapy outcome is thus the peak of a pyramid built upon these instrumentation layers. If the root-level hardware lacks optimization, the therapeutic potential of even the most advanced MNPs is effectively bottlenecked. Consequently, establishing a systematic understanding of RF generator and coil design is essential for advancing MH toward more standardized, high-precision instrumentation.

**Fig. 1:**
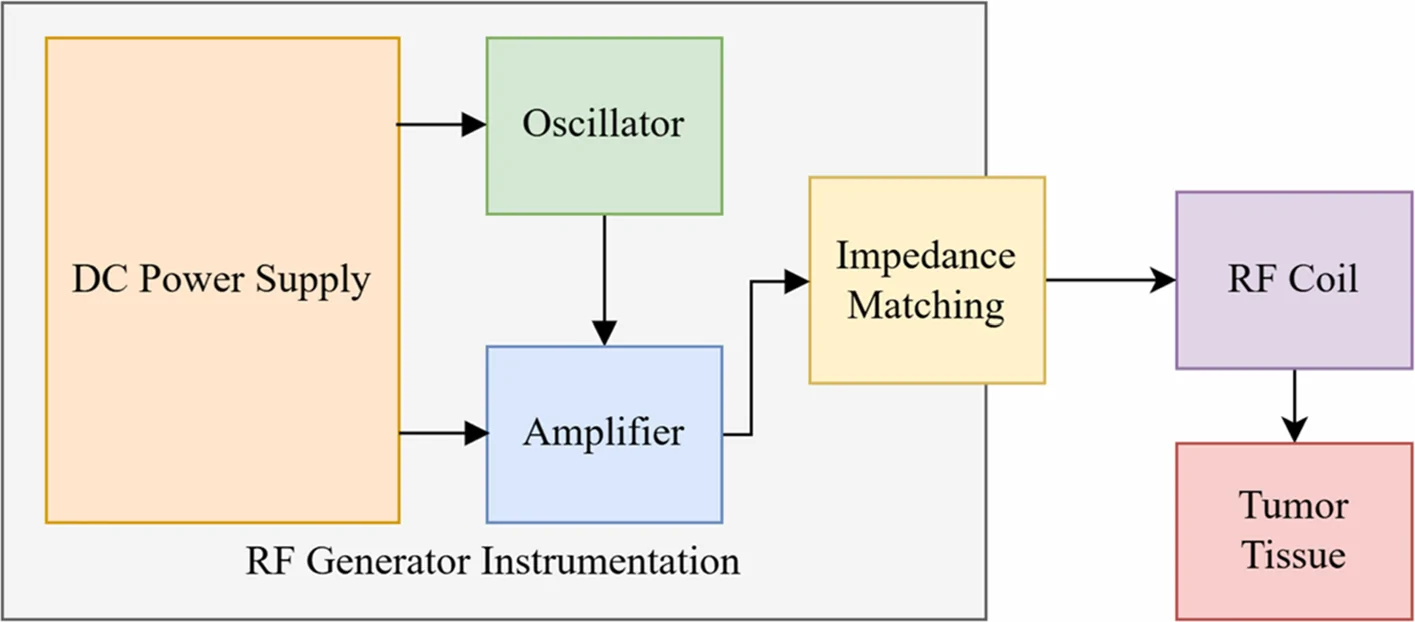
Generalized hardware block diagram of a modern MH system. Schematic showing the electrical energy generation by the RF generator instrumentation, transfer to the RF coil, and subsequent induction of electromagnetic heating within the tumor tissue

Because these hardware elements jointly determine the therapeutic fidelity and clinical safety of the MH pipeline, a structured approach is required to understand how design choices propagate from the power source to the target tissue. This review therefore separates system components into two mutually dependent domains, emphasizing that improvements in one domain often impose constraints or open opportunities in the other. The goal of this work is to provide an engineering-focused overview of recent advances in MH instrumentation, with particular emphasis on two critical system components (Fig. 1):

1. RF generator instrumentation: consisting of the DC power supply, oscillator topologies, power amplifier stages, and impedance matching networks. These elements govern the frequency selection, power delivery, and the stability necessary to maintain tank circuit resonance for efficient power transfer while driving the system reliably under dynamic loading.
2. RF coil design and thermal management: consisting of geometry, inductance, and cooling strategies. These factors determine the spatial intensity and distribution of the induced magnetic field while ensuring that operational frequencies remain safely below the coil’s self-resonant frequency. Furthermore, advanced cooling strategies are vital for maintaining long-term thermal stability and therapeutic selectivity by suppressing parasitic resistive heating, thereby ensuring that thermal delivery remains purely MNP-mediated.

**Fig. 2:**
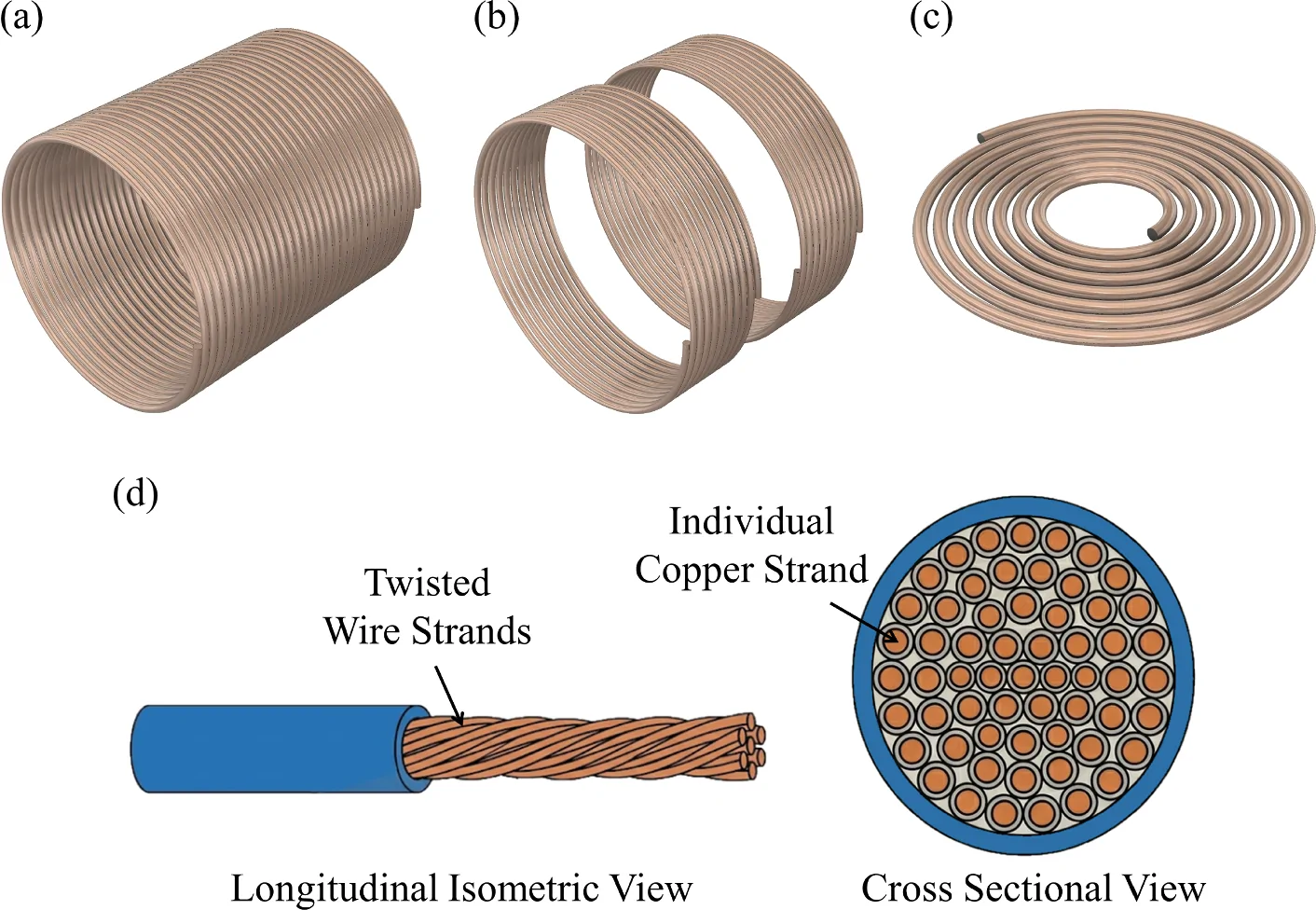
Schematics of widely utilized coil geometries and construction: **a** a solenoid coil consisting of multiple turns wound along a cylindrical axis; **b** a dual-coil configuration representing both Helmholtz and Maxwell topologies, where two identical coaxial circular coils are separated axially to provide either high field homogeneity (Helmholtz: d=R, in-phase) or a linear field gradient (Maxwell: $d=\sqrt{3}R$, anti-parallel); **c** a planar coil with turns arranged in a single plane; and **d** Litz wire to minimize RF power losses

Instead of offering an exhaustive survey, this review highlights a selection of influential and technically significant studies [15–19] that illustrate the current capabilities and limitations of modern MH instrumentation. These studies were selected according to the following criteria: (i) architectural distinctiveness, defined as a documented difference in at least one of the seven hardware stages evaluated in this review (DC power supply, oscillator, amplifier, impedance matching, coil geometry, inductance, and cooling strategy) relative to every other included system; (ii) publication in the peer-reviewed literature between 2016 and 2025, a window capturing the field’s transition from discrete analog and insulated-gate bipolar transistor (IGBT)-based architectures toward digitally controlled, Silicon Carbide (SiC)-based, and industrial-derived designs; (iii) presence of a physically constructed and experimentally validated hardware prototype, excluding simulation-only or purely theoretical designs; (iv) sufficient component-level documentation (manufacturer, model number, or equivalent specification) to support the ordinal scoring framework defined in “Framework for system-level evaluation of MH architectures" section; and (v) variation in electromagnetic interface, encompassing diverse RF coil geometries including solenoidal, Helmholtz, Maxwell, and planar configurations (Fig. 2a–c) implemented with both regular and Litz wire (Fig. 2d). By construction, the selected systems span this representative cross-section of coil topologies and winding strategies rather than concentrating on incremental variations of a single geometry. It should be stated explicitly that this work is not conducted as a systematic review in the formal methodological sense (e.g., following PRISMA guidelines), and does not employ a documented database search protocol, a defined set of search keywords, a formal literature-flow diagram, or a reported count of records identified, screened, and excluded. Rather, the five systems evaluated in the ordinal framework were purposively selected according to the architectural-distinctiveness, publication-window, hardware-validation, documentation, and geometric-diversity criteria enumerated above, an approach appropriate to a structured, criterion-based engineering review of representative design archetypes, but distinct from the exhaustive, reproducible retrieval methodology a systematic review requires. This work is accordingly characterized throughout as a structured, critical engineering review of MH instrumentation trade-offs, rather than as a systematic review.

Systems originating from overlapping research groups—notably Zeinoun et al.[17] and Garcia et al.[18]—were retained as separate entries because they employ architecturally distinct oscillator stages (MCU-based versus FPGA-based PWM control, respectively) and were published five years apart, representing successive rather than duplicate design generations. Yöner et al. [20, 21] is included in this review for architectural context but is excluded from the ordinal scoring framework described in “Framework for system-level evaluation of MH architectures” section.

This also examines the integration of real-time image-guided hyperthermia to ensure spatial targeting and patient safety in clinical-scale architectures. By construction, the selected systems span a representative cross-section of the contemporary MH design space, covering high-power bench-top platforms, portable low-power units, and hybrid architectures integrating real-time temperature sensing or imaging modalities. This diversity ensures that the analysis reflects the principal system-level trade-offs encountered in MH hardware, specifically the balance between field intensity, homogeneity, and thermal stability rather than focusing on incremental variations of similar designs. While specialized commercial platforms such as the MagForce NanoActivator [22] and nanoTherics magneTherm [23] provide robust, turn-key performance for clinical-scale applications and laboratory research, this review focuses primarily on technically innovative designs that introduce new concepts, architectures, or methodological frameworks where design choices are sufficiently documented to enable system-level interpretation. Beyond these commercial platforms, the broader clinical and investigational landscape includes systems such as the MagForce F100/MFH 300F applicator with integrated thermometry [24], the only MH system to have received clinical approval to date; Stauffer et al.’s foundational work on clinical magnetic-induction heating of ferromagnetic implants [25]; the investigational New Phase Electromagnetic Induction System (EIS) and Sarah Nanoparticle platform [26]; and Solenic Medical’s SOLA2 AMF Knee System, an FDA-authorized adjacent AMF implant-heating platform whose underlying solenoid-coil approach to implant heating is described in related engineering work [27]. These systems are not included in the ordinal scoring framework, as they either lack the component-level documentation required by the selection criteria defined above or are designed for a substantially different scale and regulatory context than the pre-clinical research prototypes evaluated in this review; they are noted here to situate the reviewed systems within the wider landscape of MH instrumentation development. Several important topics, such as MNP synthesis protocols, advanced bio-heat modeling, and clinical oncology results, are outside the scope of this review but are referenced when relevant to hardware interpretation. The intention is to provide a structured engineering perspective on the MH hardware design space, enabling meaningful comparison of representative architectures without diluting the analysis across a large number of incrementally similar or proprietary systems.

Understanding how MH hardware and system-level design choices affect heating efficiency requires a formal link between the applied AMF, the magnetic properties of the MNPs, and the resulting thermal energy dissipation. This relationship is quantified by the power loss density (*P*) [28]:

1 $$\begin{aligned} P = \pi \mu _{0}\chi ''f H^{2}, \end{aligned}$$

wherein *P* is the power dissipated per unit volume, $$\mu _0$$ is the permeability of free space, $\chi^{\prime \prime }$ is the imaginary component of the magnetic susceptibility, *f* is the operating frequency, and *H* is the magnetic field intensity. Unlike the induction heating of bulk metals, MH efficiency is highly frequency-dependent. By expanding $\chi^{\prime \prime }$ to account for the relaxation time (τ) of the MNPs within the Linear Response Theory (LRT) framework [29], the relationship is further refined:

2 $$\begin{aligned} \chi '' = \chi _{0}\frac{2\pi f\tau }{1+(2\pi f\tau )^{2}}. \end{aligned}$$

This expansion illustrates that maximum power dissipation occurs when the hardware’s operating frequency is synchronized with the relaxation mechanisms of the magnetic material ($2\pi f\tau = 1$). In this expression, $\chi _{0}$ represents the static susceptibility, which defines the material’s equilibrium magnetic response. While the fractional term determines the frequency-dependent synchronization, the static susceptibility dictates the overall magnitude of energy absorption that the instrumentation must facilitate. It is important to note that LRT, as expressed in Eq. (2), is strictly valid only for MNPs with strong magnetic anisotropy driven at low field amplitudes, where the induced magnetization response remains approximately linear with the applied field [30]. For weakly anisotropic MNPs, or for the higher field amplitudes typically required to achieve therapeutically relevant SLP, this linearity assumption breaks down and the hysteresis loop area, rather than the LRT-derived susceptibility, must be calculated using dynamic Stoner–Wohlfarth model-based theories (SWMBTs), which account for the full non-linear magnetization reversal process under a driven alternating field [30]. Both frameworks are therefore relevant to MH instrumentation design: LRT provides a tractable, closed-form description of the frequency-dependent susceptibility appropriate for strongly anisotropic MNPs operated within the low-field regime characteristic of several of the systems reviewed here, whereas dynamic Stoner–Wohlfarth-based models are more appropriate for characterizing hardware operating at higher field amplitudes or with weakly anisotropic particles, where hysteretic losses dominate. The choice between these frameworks depends on the specific field amplitude and MNP anisotropy regime targeted by a given instrumentation architecture, underscoring that no single analytical model is universally applicable across the full design space surveyed in this review.

Consequently, the choice of oscillator stage and its ability to maintain or tune frequency is a critical determinant of system performance. However, hardware design is commonly constrained by the Atkinson–Brezovich limit [31, 32], a widely used conservative threshold representing the physiological limit for non-specific eddy current heating in healthy tissue:

3 $$\begin{aligned} f\times H<4.85\times 10^8 \,\mathrm{Am}^{-1}\mathrm{s}^{-1}. \end{aligned}$$

It should be noted that the Atkinson–Brezovich limit, while widely adopted as a conservative design constraint, is not a strict physical bound on hardware operation; several MH systems, including some reviewed in this work, are deliberately operated beyond this threshold under the looser Hergt–Dutz criterion ($f\times H<5\times 10^{9} \,\mathrm{A}\mathrm{m}^{-1}\mathrm{s}^{-1}$), which permits substantially higher combined frequency-field products [33]. This creates a fundamental instrumentation trade-off: higher frequencies (*f*) and field strengths (*H*) enhance the SLP, but are constrained by this safety ceiling and the resulting increase in hardware complexity, such as the requirement for advanced cooling to manage the high reactive power in the tank circuit. The optimization of the RF generator and coil interface is therefore a balancing act between maximizing therapeutic energy transfer and maintaining operation within these strictly defined safety and efficiency limits.

It should be emphasized that the f×H product constraint discussed above addresses a single, historically established criterion for non-specific eddy current heating and does not, by itself, constitute a complete characterization of MH system safety. A more complete safety assessment requires two conceptually distinct considerations. The first, electromagnetic exposure safety, concerns the physical interaction of the applied field with the patient’s body as a whole: the spatial distribution of the field, patient and body-region geometry, the induced electric field, and the resulting eddy currents in conductive tissue. The second, thermal safety, concerns the biological consequence of this electromagnetic exposure: the resulting tissue losses, the possibility of local hot spots arising from field inhomogeneity or tissue heterogeneity rather than from the nominal f×H product alone, and the actual thermal dose delivered to both target and non-target tissue over the course of treatment. The f×H criterion (and its less conservative Hergt–Dutz counterpart) addresses only a coarse, whole-body proxy for the electromagnetic-exposure side of this assessment; it does not account for spatially localized field inhomogeneities, patient-specific anatomy, or the resulting thermal dose distribution, each of which can produce localized safety concerns not captured by a single scalar frequency-field product. We adopt this electromagnetic-exposure/thermal-safety distinction throughout the remainder of this review and revisit it in “Building the next generation of MH hardware" section in the context of eddy-current mitigation and spatial field confinement strategies.

## Framework for system-level evaluation of MH architectures

Evaluating MH hardware at the system level is challenging due to the lack of standardized reporting of key performance indicators such as SLP, field homogeneity, and thermal stability across the literature. As a result, direct quantitative comparison between heterogeneous architectures is often not feasible, and system-level trade-offs are typically discussed in a qualitative or application-specific manner. To address this limitation, this work introduces a structured evaluation framework based on physically grounded proxy metrics, enabling consistent and reproducible comparison of diverse MH designs. Rather than relying on incomplete or non-uniformly reported numerical values, the proposed framework maps key design parameters onto ordinal scales that preserve relative differences without introducing false precision. This approach allows system architectures to be interpreted within a unified design space defined by generation performance and transduction fidelity, thereby providing a practical basis for analyzing trade-offs across diverse MH implementations.

It is important to state explicitly the scope of ’system-level’ optimization as employed in this review. The evaluation framework developed here addresses the instrumentation chain from the energy source through to the generated magnetic field, encompassing the RF generator, impedance-matching network, and coil/magnetic system and does not extend to the downstream physiological chain linking the generated field to nanoparticle magnetic response, heat generation, heat transfer, tissue temperature, thermal dose, and ultimate biological effect. Consequently, the $G_{ordinal}$ and $C_{ordinal}$ metrics proposed here should be interpreted as engineering rankings of instrumentation architecture sophistication and design completeness, rather than as demonstrated measures of therapeutic effectiveness. A system achieving a higher ranking under this framework is not thereby established to deliver a greater or more effective thermal dose to a target volume; such a determination would additionally require consideration of the fraction of delivered energy absorbed by the nanoparticle sample, the volume over which this energy is deposited, and quantities such as SLP/specific absorption rate (SAR)/intrinsic loss power (ILP), none of which are consistently reported for the systems considered.

Beyond their inconsistent reporting, SLP/SAR/ILP values are also strongly dependent on the specific nanoparticle formulation, concentration, and sample geometry employed in a given study, factors that are independent of the instrumentation architecture this framework is designed to isolate and compare. Incorporating such values into a hardware-focused ordinal ranking, even where reported, would therefore risk conflating genuine architectural performance differences with differences attributable to the nanoparticle system tested rather than the instrumentation itself, a confound that a standardized, common-nanoparticle testing protocol across systems would be required to resolve. For this reason, SAR/SLP/ILP-linked outcomes are treated in this review as an important complementary dimension for future, purpose-designed comparative studies, rather than as parameters to be incorporated into the present architecture-focused ordinal framework. This distinction is illustrated directly in “DC power supply" section: the highest-power system evaluated here (Attaluri et al. [19], 120 kW) achieves the highest $R_{psu}$ score under this framework’s power-headroom criterion, yet is explicitly characterized in that section as ill-suited to most practical MH applications given its cost, footprint, and lack of MH-specific tuning, underscoring that a high ranking on a given instrumentation axis does not, by itself, imply superior therapeutic suitability.

It should also be noted that the scope of this review is limited to instrumentation generating conventional, linearly oscillating AMF, consistent with the great majority of the MH instrumentation literature. Rotating magnetic field (RMF) systems, in which the field vector’s direction, not only its amplitude, varies in time, represent a distinct electromagnetic excitation strategy with a growing, dedicated body of instrumentation and nanoparticle-response literature. Because RMF generator architectures (typically multi-phase excitation with distinct magnetic-circuit topologies) differ fundamentally from the single-axis AMF architectures evaluated in this review’s ordinal framework, a rigorous comparative treatment of RMF instrumentation is considered outside the scope of the present review and is discussed separately in “Building the next generation of MH hardware" section as an important direction for future engineering-focused review work.

For the RF generator instrumentation performance, a metric is defined to capture the overall system-level capabilities of the generator hardware. Each key component is represented by a single, clearly defined performance aspect: the DC power supply stage (power headroom), the oscillator stage (frequency tunability), the amplifier stage (harmonic fidelity), and the impedance matching stage (coupling efficiency). Each system is ranked relative to the others for that aspect, and a weighted sum of the ranks forms the overall RF generator instrumentation performance metric G_perf_:

4 $$\begin{aligned} G_\mathrm{perf} = w_{1}\cdot R_\mathrm{psu} + w_{2}\cdot R_\mathrm{osc} + w_{3}\cdot R_\mathrm{amp} + w_{4}\cdot R_\mathrm{imp}, \end{aligned}$$

where ${R}_{\mathrm{psu}}$, ${R}_{\mathrm{osc}}$, ${R}_{\mathrm{amp}}$, and ${R}_{\mathrm{imp}}$ are the ordinal ranks for the DC power supply, oscillator, amplifier, and impedance matching, respectively. The weighting coefficients $w_{i}$ allow flexible adjustment of the relative contribution of each component; in this work, equal weighting is adopted for all variables to ensure a neutral and unbiased comparison.

Each of these four axes is ranked according to a criterion specific to its available reporting in the literature. ${R}_{\mathrm{psu}}$ is ranked directly by each system’s rated power delivery (voltage × current, or manufacturer-rated peak power where a direct product is not reported), as this value is consistently documented across all five systems. ${R}_{\mathrm{osc}}$ is ranked by each system’s reported frequency tuning range (in kHz), reflecting the breadth of spectral adjustability available to the operator or control system; where a system’s frequency is fixed or determined entirely by external LC components (e.g., self-resonant designs), the tuning range is taken as the reported operational spread across documented configurations. ${R}_{\mathrm{amp}}$ and ${R}_{\mathrm{imp}}$, for which the literature does not consistently report quantitative measures such as total harmonic distortion or coupling efficiency, are ranked according to an architectural-tier ordering grounded in established power-electronics and RF-matching theory: for ${R}_{\mathrm{amp}}$, linear Class AB amplification is ranked above SiC-based full-bridge switching, which is ranked above silicon IGBT-based full-bridge switching, which is ranked above amplifier stages merged with the oscillator in a self-resonant topology [34]; for ${R}_{\mathrm{imp}}$, matching networks providing simultaneous reactance cancellation and resistive transformation are ranked above single-reactance-cancellation networks, which are ranked above transformer-based magnitude matching, which is ranked above resonant-capacitor-only matching, which is ranked above the absence of a dedicated matching stage [35]. This architectural-tier approach is adopted specifically because the reviewed literature does not consistently report the directly measured quantities that would provide a more rigorous basis for this ranking, namely total harmonic distortion (THD), the amplitudes of individual harmonics, the full signal spectrum, and frequency and amplitude stability under load. Where such measurements are reported for a given system, they would provide a strictly preferable basis for ranking harmonic fidelity; in their general absence across the reviewed literature, architectural tier is retained as the most consistently available proxy, with the explicit caveat that amplifier class alone does not fully determine the quality of the magnetic field delivered to the nanoparticle sample. It should further be noted that THD alone, while a substantial improvement over architectural classification, is not in itself a complete characterization of MH generator spectral output: Yöner et al. [20] demonstrate that a single aggregate THD figure can obscure clinically relevant differences in harmonic content relevant to inductive safety, and propose a harmonic residual index as a more discriminating, standardized spectral-purity metric for MH RF generators. This indicates that even where quantitative harmonic data are available, the choice of summary metric itself requires care, reinforcing our decision to treat architectural tier as a necessarily coarse proxy rather than to substitute an incomplete quantitative measure in its place.

One additional system, Yöner et al. [20, 21], developed by the authors of this review, is presented in Tables 2 and 3 for technological context and comparison of architectural choices, but is deliberately excluded from the ordinal scoring framework and from the composite $G_{\mathrm{ordinal}}$ and $C_{\mathrm{ordinal}}$ metrics. This exclusion is made to preserve the objectivity of the comparative framework, as scoring a system authored by the reviewers themselves against externally developed systems would introduce an unavoidable conflict of interest regardless of the outcome.

The resulting $G_{\mathrm{perf}}$ values are then ranked across systems to assign an overall ordinal score $G_{\mathrm{ordinal}}$:

5 $$\begin{aligned} G_{\mathrm{ordinal}} = \mathrm{rank}(G_{\mathrm{perf}})\in \{1, 2, \ldots , k\}, \end{aligned}$$

where *k* is the total number of systems. To address performance ties without introducing artificial stratification, the system utilizes a mean-ranking protocol. When *N* studies exhibit identical $G_{\mathrm{perf}}$ metrics, they are collectively assigned the arithmetic mean of the ordinal positions ($G_{\mathrm{ordinal}}$) they would otherwise span. For instance, if two entries compete for the top tier, they both receive a score of 4.5, the average of the positions 4 and 5, thereby maintaining a balanced and objective evaluation of shared design archetypes. Following the evaluation of the RF generator instrumentation, the RF coil design and thermal management performance is assessed using metrics that capture the physical interaction between the hardware and the target tissue. For the electromagnetic interface and thermal regulation performance, a metric is defined to capture the overall capabilities of the coil-level hardware. Each key component is represented by a single, clearly defined performance aspect: the coil geometry (magnetic field homogeneity), the coil inductance (current to magnetic field conversion efficiency), and the cooling strategy (heat dissipation capacity).

The magnetic field homogeneity represents the spatial uniformity of the induced magnetic field within the target volume, which determines the precision and predictability of the thermal dose. The coil inductance is used as a proxy for coil electromagnetic architecture, subject to the important caveats detailed below. It should be noted that inductance is not, by itself, a measure of field-generation effectiveness: as a first approximation, the magnetic field inside a coil depends primarily on system geometry, turn count, and drive current, whereas inductance principally defines the relationship between current and magnetic flux, and the energy stored in the magnetic field. At a given operating frequency, an increase in inductance correspondingly increases the coil’s reactive impedance (${XL} = 2\pi {fL}$), which raises the voltage the RF generator must supply to sustain a given drive current; a higher-inductance coil is therefore not inherently a more effective or more energy-efficient design. Nor does inductance alone determine the coil’s quality factor: both the magnetic field and Q also depend on coil geometry, winding configuration, and AC wire resistance, none of which are consistently reported across the surveyed literature. Coil geometry is accounted for independently within this framework through $R_{\mathrm{geo}}$ (defined below), so the two axes are intended to be interpreted jointly rather than $R_{L}$ being read as a standalone measure of field-generation capability. Because sufficient wire-resistance data are unavailable across the reviewed systems to calculate quality factor directly, inductance is retained here not as a performance indicator but as a descriptor of coil electromagnetic architecture and reactive design burden—the most consistently and quantitatively reported axis available for this purpose across the reviewed literature. The robustness of this axis against introducing systematic bias into the overall coil-performance ranking is assessed quantitatively in the sensitivity analysis below (Table 1).

A more direct basis for comparison would, in principle, be provided by quantities such as the active power delivered to the coil, coil resistive losses, overall system energy efficiency, coil quality factor (*Q*), and directly measured magnetic-field amplitude. The availability of each of these quantities was therefore assessed across the five ranked systems (Table 1). None of the five systems reports the complete set of these quantities, and the majority report none of them beyond a nameplate or rated power figure. This scarcity is not incidental to the present analysis; rather, it is itself among the review’s principal findings, and is the direct methodological reason an ordinal, proxy-based comparison framework is adopted here rather than a framework built on direct quantitative performance metrics. Restricting this review’s scope to systems reporting the full set of quantities identified above would have precluded meaningful comparison of the architecturally representative and well-documented systems considered, effectively preventing the present comparative analysis from being conducted at all.

Finally, the heat dissipation capacity is evaluated based on the implementation of cooling strategies, predominantly categorized as either convective air cooling or circulating liquid cooling, designed to mitigate resistive (Joule) heating of the coil. This parameter captures the system’s ability to maintain a stable thermal environment, thereby increasing/maintaining therapeutic selectivity by ensuring that the measured temperature rise is strictly MNP-mediated. Efficient dissipation prevents conductive heat transfer from the hardware to the tissue, which is essential for preserving the spatial accuracy of the treatment and preventing non-specific thermal damage to surrounding tissue.

It is important to state explicitly that this axis, and the $R_{\mathrm{cs}}$ score derived from it, characterizes thermal management of the hardware, specifically, dissipation of resistive heating within the coil itself, and does not characterize thermal management of the therapeutic volume, i.e., active control of the temperature actually achieved within the target tissue or sample. These are two distinct physical problems: the former concerns preventing parasitic, non-MNP-mediated heat from confounding the coil’s electromagnetic performance and the interpretation of experimental results, while the latter concerns the closed-loop regulation of the therapeutic thermal dose itself, typically achieved through temperature sensing and feedback control of the RF generator’s output rather than through coil cooling. Systems in this review that implement the latter, such as the PI-controlled temperature regulation described for Chatterjee and Pal [15] in “Auxiliary hardware and multimodal integration", are discussed there as auxiliary hardware rather than folded into the $R_{\mathrm{cs}}$ axis, precisely because hardware-directed cooling and therapeutic-volume temperature control address different physical quantities and are not interchangeable within a single ’cooling performance’ parameter.

Each of these three axes is ranked according to a criterion specific to its available reporting in the literature. $R_{\mathrm{geo}}$ is ranked by the documented degree of deliberate, purpose-engineered spatial field control achieved by each coil topology, as established in prior electromagnetic modeling literature [36, 37], rather than by field homogeneity alone. Dual-coil configurations governed by precise, analytically derived coil-spacing relationships, Helmholtz (d=R, in-phase, engineered for maximal central field homogeneity) and Maxwell ($d=\sqrt{3}R$, anti-parallel, engineered for a maximally linear field gradient) represent the highest tier of deliberate spatial field engineering considered in this review: each achieves a specific, well-characterized field objective through precise geometric control, rather than approximating a field profile as an incidental property of a simpler winding. Neither configuration is ranked above the other in a general sense, as homogeneity and gradient generation serve distinct and non-comparable therapeutic objectives (“Geometry" section); both are instead ranked jointly above single-coil geometries whose spatial field behavior arises as an inherent, less deliberately engineered property of the winding itself. Among these single-coil geometries, solenoids, which provide central-region uniformity with boundary-region decay, are ranked above ellipsoidal planar geometries, which are ranked above single-plane planar geometries, reflecting the progressively higher field gradients associated with reduced coil symmetry.

This ordering reflects a single, explicitly defined criterion: the degree of deliberate, purpose-engineered spatial field and gradient control achieved by each coil geometry, and should not be interpreted as a universal statement of geometric superiority. The Helmholtz and Maxwell configurations therefore occupy the highest tier considered here, as both employ analytically defined coil-spacing and current–direction relationships to deliberately shape the spatial magnetic field: the Helmholtz configuration is engineered to maximize central-region field uniformity, whereas the Maxwell configuration is engineered to generate a maximally linear field gradient. Neither configuration is ranked above the other in a general sense, as they represent distinct forms of deliberate spatial field engineering serving different therapeutic objectives. They are therefore ranked jointly above single-coil geometries, for which the spatial field profile and associated gradients arise primarily from the inherent symmetry and winding geometry rather than from a specifically engineered multi-coil field relationship. Among the single-coil geometries, solenoids, which provide comparatively controlled central-region fields with gradual boundary-region decay, are ranked above ellipsoidal planar geometries, which are ranked above single-plane planar geometries, reflecting the progressively reduced geometric symmetry and consequently less controlled spatial field distribution. The geometry ranking employed here therefore represents a criterion-specific assessment of spatial field-control capability; a different ordering could result under an alternative therapeutic objective, such as deep-tissue penetration, focal targeting, or field-free region (FFR) localization (“Building the next generation of MH hardware"). $R_{\mathrm{geo}}$ should accordingly be interpreted as a criterion-specific rather than universal geometric ranking. $R_L$ is ranked directly by each system’s reported coil inductance value (in μH), as this value is consistently documented across all five systems. $R_{\mathrm{cs}}$ is ranked according to whether a system’s cooling strategy directly targets the RF coil: systems in which the coil itself relies solely on passive convective air cooling are assigned a shared baseline tier, regardless of whether liquid cooling is implemented elsewhere in the system (e.g., for thermal isolation of the MNP sample rather than for coil cooling); systems implementing active liquid cooling directed at the coil itself are assigned the highest tier. As with the RF generator axes, systems for which a given axis’s underlying value is not reported in the source literature are excluded from ranking on that axis only, rather than assigned a default score.

Applying the same weighting and ranking logic established for the RF generator instrumentation performance, the overall RF coil design and thermal management performance metric $C_{\mathrm{perf}}$ is expressed as:

6 $$\begin{aligned} C_{\mathrm{perf}} = w_{1}\cdot R_{\mathrm{geo}} + w_{2}\cdot R_{L} + w_{3}\cdot R_{\mathrm{cs}}, \end{aligned}$$

where $R_{\mathrm{geo}}$, $R_{L}$, and $R_{\mathrm{cs}}$ are the ordinal ranks of each system for the geometry, inductance, and cooling strategy, respectively. The resulting $C_{\mathrm{perf}}$ values are then ranked to assign an overall ordinal score $C_{\mathrm{ordinal}}$:

7 $$\begin{aligned} C_{\mathrm{ordinal}} = \mathrm{rank}(C_{\mathrm{perf}}) \in \{1, 2, \ldots , k\}. \end{aligned}$$

Finally, because exact numerical values for SLP or field gradients are not consistently reported in the literature, all variables contributing to the proposed metrics are assigned ordinal ranks (1–5) across the available designs. This procedure ensures that relative differences in design sophistication such as the transition from basic, air-cooled solenoids to precision-engineered, liquid-cooled Maxwell or Helmholtz configurations are preserved without relying on absolute measurements that vary based on experimental conditions.

To assess the robustness of the ranking outcomes to the choice of equal weighting, a sensitivity analysis was performed by recomputing $G_{\mathrm{perf}}$ and $C_{\mathrm{perf}}$ under two alternative weighting schemes for each metric (Table 1), in addition to the equal-weighting baseline. For $G_{\mathrm{perf}}$, a power-emphasis scheme ($w_{\mathrm{psu}}=w_{\mathrm{amp}}=2$, $w_{\mathrm{osc}}=w_{\mathrm{imp}}=1$) and an agility-emphasis scheme ($w_{\mathrm{osc}}=w_{\mathrm{imp}}=2$, $w_{\mathrm{psu}}=w_{\mathrm{amp}}=1$) were tested. For $C_{\mathrm{perf}}$, a geometry-emphasis scheme ($w_{\mathrm{geo}}=2$, $w_L=w_{\mathrm{cs}}=1$) and a cooling-emphasis scheme ($w_{\mathrm{cs}}=2$, $w_{\mathrm{geo}}=w_L=1$) were tested. In all cases, the resulting $G_{\mathrm{ordinal}}$ and $C_{\mathrm{ordinal}}$ rank orderings were identical to those obtained under equal weighting: Chatterjee and Pal [15] < Valadez et al. [16] < Garcia et al. [18] < Attaluri et al. [19] < Zeinoun et al. [17] for $G_{\mathrm{ordinal}}$, and Chatterjee and Pal [15] ≈ Valadez et al. [16] < Zeinoun et al. [17] ≈ Garcia et al. [18] < Attaluri et al. [19] for $C_{\mathrm{ordinal}}$. This stability indicates that the comparative conclusions drawn in this review are not an artifact of the specific weighting scheme adopted, but reflect underlying, weighting-independent differences in the reported hardware specifications across the reviewed systems. A further methodological caveat is warranted regarding the precision implied by the ordinal scale itself. With only five systems included in the ranked comparison, presenting $G_{\mathrm{ordinal}}$ and $C_{\mathrm{ordinal}}$ to a resolution of 0.5 (arising from the mean-ranking protocol for ties) risks conveying a degree of statistical precision the underlying evaluation model does not support. These values should be read as relative orderings within a small, purposively selected, non-random sample of architecturally representative systems, rather than as measurements with an implied margin of error; the numerical granularity is a property of the ranking and tie-breaking procedure adopted, not of the instrumentation itself. Correspondingly, the stability of the rank ordering across alternative weighting schemes, discussed above, should be interpreted as evidence that our comparative conclusions are robust to the specific methodological choices made in constructing this framework, not as independent evidence that a given system is physically superior as an MH instrument; the ranking remains, throughout, the output of an evaluation model adopted by the authors, rather than a direct measurement of system performance

To directly test whether the inclusion of coil inductance introduces a systematic bias into the coil-performance ranking, $C_{\mathrm{perf}}$ was additionally recomputed with the $R_{L}$ axis excluded entirely. The resulting rank structure Attaluri et al. [19] highest, Zeinoun et al. [17] and Garcia et al. [18] at a shared intermediate tier, and Chatterjee and Pal [15] together with Valadez et al. [16] at the lowest tier, is preserved under this exclusion, with only the internal ordering within the lowest tier changing. This indicates that the inclusion of inductance as a proxy axis does not materially alter the comparative conclusions of the coil-performance ranking.

**Table 1:** Sensitivity of ordinal rank assignments to alternative weighting schemes

Study Group	$G_{\mathrm{ordinal}}$			$C_{\mathrm{ordinal}}$			
	Equal	Power	Agility	Equal	Geometry	Cooling	Inductance
	(baseline)	$\mathrm{emphasis}^{a}$	$\mathrm{emphasis}^{b}$	(baseline)	$\mathrm{emphasis}^{c}$	$\mathrm{emphasis}^{d}$	$\mathrm{excluded}^{e}$
Chatterjee and Pal [15]	1	1	1	1.5	1.5	1.5	1
Valadez et al. [16]	2	2	2	1.5	1.5	1.5	2
Garcia et al. [18]	3	3	3	3.5	3.5	3.5	3.5
Attaluri et al. [19]	4	4	4	5	5	5	5
Zeinoun et al. [17]	5	5	5	3.5	3.5	3.5	3.5

Reported values are $G_{\mathrm{ordinal}}$ and $C_{\mathrm{ordinal}}$ rank positions (1–5, with 5 highest) for each system under equal weighting and two alternative weighting schemes per metric. Rank order is identical across all tested schemes for both metrics

$$^{a} w_{\mathrm{psu}}=w_{\mathrm{amp}}=2$$, $$w_{\mathrm{osc}}=w_{\mathrm{imp}}=1$$

$$^{b} w_{\mathrm{osc}}=w_{\mathrm{imp}}=2$$, $$w_{\mathrm{psu}}=w_{\mathrm{amp}}=1$$

$$^{c} w_{\mathrm{geo}}=2$$, $$w_L=w_{\mathrm{cs}}=1$$

$$^{d} w_{\mathrm{cs}}=2$$, $$w_{\mathrm{geo}}=w_L=1$$

$^{e} w_{\mathrm{geo}}=w_{\mathrm{cs}}=1$, $R_{L}$ excluded from $C_{\mathrm{perf}}$ entirely

The ordinal rank assignments underlying $G_{\mathrm{perf}}$ and $C_{\mathrm{perf}}$ were determined by both authors independently applying the criteria defined above to the source literature prior to any discussion, followed by cross-checking of all assignments and resolution of any discrepancies by consensus reference to the original source data. Because the majority of ranking criteria in this framework are anchored either to directly reported numerical values ($R_{\mathrm{psu}}$, $R_{\mathrm{osc}}$, $R_L$) or to established, citation-grounded architectural-tier orderings ($R_{\mathrm{amp}}$, $R_{\mathrm{imp}}$, $R_{\mathrm{geo}}$, $R_{\mathrm{cs}}$; see criteria above), rather than to unconstrained subjective judgment, the scope for inter-rater disagreement within this framework is inherently limited compared to fully qualitative scoring schemes.

It is worth stating explicitly which axes in this framework are derived from directly reported quantitative values and which rely on architectural-tier judgment, since this distinction bears on both the precision of the resulting ranks and the feasibility of formal uncertainty quantification. $R_{\mathrm{psu}}$, $R_{\mathrm{osc}}$, and $R_{L}$ are ranked directly from quantitative values reported in the source literature (rated power in W, tuning range in kHz, and inductance in μH, respectively; see Tables 2 and 3), and are accordingly the axes for which the ordinal ranking most closely tracks an underlying continuous measurement. $R_{\mathrm{amp}}$, $R_{\mathrm{imp}}$, $R_{\mathrm{geo}}$, and $R_{\mathrm{cs}}$, by contrast, are necessarily ranked by architectural-tier criteria because the literature does not consistently report the underlying continuous quantities (THD, coupling efficiency, field-homogeneity measurements, and coil-surface temperature rise, respectively) that would permit a quantitative ranking of these axes. A formal uncertainty analysis, such as a confidence interval or bootstrap estimate, is well suited to a ranking derived from measured values with associated measurement error, but has no direct analogue for a ranking derived from categorical architectural judgment, where the relevant uncertainty is instead the possibility of disagreement over the tier ordering itself, rather than measurement noise around a reported number. We therefore report this quantitative/architectural distinction explicitly, in place of a formal uncertainty analysis, as the most transparent way to convey where this framework’s rankings rest on firmer versus softer evidentiary ground.

## RF generator instrumentation

The RF generator instrumentation serves as the primary power delivery and orchestration stage for MH. The system’s operation begins with a dedicated DC power supply stage that provides regulated DC power, drawing from stable and high-capacity sources to meet the significant energy demands of the oscillator and amplifier stages, respectively, while the control and sensing circuitry often operate on independent, low-voltage regulated rails to ensure signal integrity and prevent electromagnetic interference. This DC power feeds the oscillator unit, which establishes the system’s fundamental operating frequency and regulates the excitation signal characteristics such as shape and duty cycle [38, 39]. Depending on the architecture, this regulation is achieved either dynamically through digital control units communicating with the driver modules, or via the inherent feedback mechanisms of analog, self-resonant oscillator circuits. This precise, low-power signal is then fed to the amplification stage, ranging from high-power bridge inverters to Class AB amplifiers, which converts the signal into the high-amplitude alternating current (AC) required for induction. High-power output is subsequently conditioned through an impedance matching network; for sinusoidal waves, this stage ensures maximum power transfer by compensating for the coil’s inductive reactance, while in non-sinusoidal or pulsed waves, it serves to optimize the coupling between the generator’s switching bridge and the inductive load [40].

Successfully balancing these complex orchestration, power, and signal conditioning functions across high-power instrumentation leads to the distinct design archetypes summarized in Table 2. The application of the developed scoring framework to these architectures reveals a clear divergence in hardware performance ($G_{\mathrm{ordinal}}$), particularly regarding power headroom and frequency agility. In terms of raw energy orchestration, Attaluri et al. [19] (AMF LifeSystems 120 kW industrial induction system) achieves the highest DC power supply score among the ranked systems ($R_{\mathrm{psu}} = 5$) by providing a 120 kW industrial-grade stage capable of meeting the massive current demands of large-scale RF coils. Furthermore, regarding the precise control of signal characteristics for advanced heating protocols, Garcia et al.’s [18] architecture utilizing a field-programmable gate array (FPGA)-based PWM controller demonstrates superior performance in the oscillator stage ($R_{\mathrm{osc}} = 5$), offering the widest documented tuning range and finest timing resolution among the ranked systems.

Conversely, the evaluation highlights a shift toward high-fidelity, low-distortion strategies among the ranked designs. Zeinoun et al. [17] and Garcia et al. [18] share the highest harmonic fidelity score ($R_{\mathrm{amp}} = 4.5$) by utilizing SiC-MOSFET-based full-bridge inverters, which achieve substantially higher switching frequencies and cleaner spectral output than the IGBT- or self-resonant-based alternatives. Meanwhile, Attaluri et al.’s [19] internal, water-cooled capacitor banks achieve the highest coupling-efficiency score ($R_{\mathrm{imp}} = 5$), reflecting both a single-reactance-cancellation matching topology and active thermal management not documented for the other systems in this tier. For additional technological context, Yöner et al. [20] employs a Class AB amplifier and a high-pass L-network, architectural choices associated with high harmonic fidelity and simultaneous reactance-and-resistance matching, respectively, though this system is excluded from the ranked comparison for the reasons described in “Framework for system-level evaluation of MH architectures” section. These scoring variations underscore the highly specialized nature of contemporary RF generator instrumentation, where design priorities ranging from high-throughput industrial induction to high-precision localized hyperthermia research are strictly aligned with the specific requirements of the intended experimental environment.

**Table 2:** Functional component choices by each significant study group for the MH RF generator and instrumentation

${\text{Study groups}}^{{G_{\mathrm{ordinal}}}}$	DC power R_psu_	Oscillator R_osc_	Amplifier R_amp_	Impedance R_imp_
$\mathrm{Chatterjee and Pal}^{\boldsymbol{1}}$ [15]	Scientific Mes-Technik PSA40–40 M/s 40 V/40 A [41]$^{\boldsymbol{1}}$	IRF540 MOSFET based half-bridge Mazzilli inverter [42]$^{\boldsymbol{3}}$	IRF540 MOSFET based half-bridge Mazzilli inverter$^{\boldsymbol{1}}$	Parallel resonant capacitor$^{\boldsymbol{2}}$
Valadez et al.$^{\boldsymbol{2}}$ [16]	Custom-built buck converter 50–200 V/50 A$^{\boldsymbol{3}}$	UCC3895DW model PWM controller [43]$^{\boldsymbol{1}}$	FF150R12RT4 model IGBT based full-H bridge [44]$^{\boldsymbol{3}}$	Dual-primary transformer$^{\boldsymbol{3}}$
Garcia et al.$^{\boldsymbol{3}}$ [18]	500 V/15 A no-brand DC power supply$^{\boldsymbol{2}}$	FPGA based PWM controller$^{\boldsymbol{5}}$	Full bridge inverter with SiC MOSFETs$^{\boldsymbol{4.5}}$	No impedance matching$^{\boldsymbol{1}}$
Attaluri et al.$^{\boldsymbol{4}}$ [19]	AMF LifeSystems 120 kW industrial induction system [45]$^{\boldsymbol{5}}$	AMF LifeSystems 120 kW industrial induction system$^{\boldsymbol{2}}$	AMF LifeSystems 120 kW industrial induction system$^{\boldsymbol{2}}$	Internal capacitor banks of the induction system$^{\boldsymbol{5}}$
Zeinoun et al.$^{\boldsymbol{5}}$ [17]	500 V/60 A no-brand DC power supply$^{\boldsymbol{4}}$	STM32-F446RE MCU based PWM controller [46]$^{\boldsymbol{4}}$	Full bridge inverter with SiC MOSFETs$^{\boldsymbol{4.5}}$	Capacitor bank$^{\boldsymbol{4}}$
Yöner et al. [20]	Two identical Mean Well UHP-750-24 model SMPS [47]	Wien-bridge oscillator	Class AB amplifier	High pass L-network

Bolded superscript numbers (1–5) indicate ordinal ranks assigned to each component for systematic evaluation among the five scored systems, with 5 representing the highest performance and 1 the lowest. Bolded superscript numbers in the first column correspond to the overall electronics performance metric $G_{\mathrm{ordinal}}$ for each system. Yöner et al. [20] is presented for technological context but is excluded from ranking to avoid a conflict of interest

Component descriptions and underlying numerical values (e.g., voltage/current ratings and frequency ranges) are as reported in the cited source publications or manufacturer datasheets. Ranks for $R_{\mathrm{psu}}$ and $R_{\mathrm{osc}}$ are computed directly from these reported values, whereas ranks for $R_{\mathrm{amp}}$ and $R_{\mathrm{imp}}$ are inferred by the authors of this review by applying the architectural-tier criteria defined in “Framework for system-level evaluation of MH architectures" section

### DC power supply

The architectural diversity among the five study groups highlights how specific DC power supply choices are dictated by the intended application’s power headroom and stability requirements. At the baseline for high-precision laboratory testing, Chatterjee and Pal [15] utilize a Scientific Mes-Technik PSA40-40 40 V/40 A [41] DC power supply. This selection prioritizes a stable and low-noise environment for fundamental signal characterization; however, the 40 V ceiling restricts the system to low-voltage, high-current experimentation, making it difficult to drive the high-impedance loads often encountered in large-scale RF coils. Shifting toward more dynamic power regulation, Valadez et al. [16] employ a custom-built buck converter capable of delivering 50 A at 50–200 V. This architecture allows the DC bus voltage to be precisely tailored to the specific voltage ratings of the subsequent oscillator and amplifier components, though the custom design introduces significant development overhead and requires robust shielding to prevent switching noise from leaking into any integrated control or monitoring electronics [48].

Even greater power capacity is observed in the high-voltage archetypes of Zeinoun et al. [17] and Garcia et al. [18], who utilize non-branded DC power supplies rated at 500 V/60 A and 500 V/15 A, respectively. Zeinoun et al. [17] leverage this high potential to overcome reactive impedance, providing substantial energy overhead for intense field generation. However, the reliance of both studies on no-brand hardware introduces risks regarding output ripple and long-term voltage stability under the extreme thermal loads of MH [49]. While Garcia et al.’s [18] 500 V supply facilitates high-voltage operation, its 15 A current ceiling creates a performance bottleneck if the system were scaled for low-impedance, high-current heating protocols.

Conversely, Attaluri et al. [19] utilize the AMF LifeSystems 120 kW [45] industrial induction system, representing the highest end of the power spectrum and, among the five ranked systems, achieving the highest documented power headroom. While this system is commercially available as off-the-shelf industrial induction equipment, it is not purpose-built or optimized for MH: its extreme cost, physical footprint, and lack of MH-specific power tuning make it a poor candidate for a dedicated, commercially viable MH product in its current form. Such an industrial-grade orchestration stage constitutes an overkill for most MH applications, as the specialized infrastructure requirements and high operational overhead make it less suitable for portable or cost-effective laboratory research. For technological context, Yöner et al. [20] adopt a modular archetype by utilizing two identical Mean Well UHP-750-24 [47] model switched mode power supply (SMPS) units, though this system is excluded from the ranked comparison for the reasons described in Sect. Framework for system-level evaluation of MH architectures. By employing industry-standard SMPS, this design prioritizes reliability and clean output over raw voltage, providing a stable energy foundation for high-precision, linear-amplification-based hyperthermia research where signal integrity and protection circuitry are paramount. In this configuration, the two supplies are connected in series, with the midpoint treated as ground, thereby providing both positive and negative voltage rails to the RF generator. This dual-rail arrangement is essential for driving linear amplification stages that require symmetric supply voltages. Although the UHP-750-24 units lack integrated control terminals, their high current capacity and proven reliability make them well-suited for continuous MH operation, while alternative supplies with built-in control features were limited by lower current ratings or reduced availability.

### Oscillator

The selection of oscillator stages across the five investigations illustrates a fundamental tension between circuit simplicity and the frequency tunability required to optimize heat dissipation in MNPs. At the lowest end of the tunability range, Valadez et al. [16] utilize a UCC3895DW [43] model phase-shift PWM controller. While this specialized chip offers stable switching for full-bridge architectures, the frequency is hard-wired at 170 kHz via external RC components, yielding no adjustable tuning range and restricting flexibility to discrete board-level modifications rather than on-the-fly adjustments.

A modest tuning range is available in the industrial solution used by Attaluri et al. [19] (AMF LifeSystems 120 kW), which emphasizes power-load stability over experimental versatility. These high-power systems are generally locked into a narrow operational bandwidth designed to protect their internal matching circuitry, restricting operation to 150 ± 10 kHz, a 20 kHz span that limits the system’s ability to probe MNP response across diverse spectral ranges.

A wider, though still hardware-constrained, tuning range is achieved by Chatterjee and Pal [15], who implement an IRF540 [42] metal-oxide-semiconductor field-effect transistor (MOSFET)-based half-bridge Mazzilli inverter. In this configuration, the excitation frequency is dictated entirely by the physical properties of the LC tank and the driven RF coil itself; because the frequency is determined by the specific hardware connected, the oscillator generated 199 kHz with one coil and 290 kHz with another, a documented operational spread of 91 kHz across configurations. Although this ensures high efficiency through automatic zero-voltage switching (ZVS), the absence of an independent clock source means frequency adjustments necessitate the manual replacement of capacitors or the RF coil itself [50], rather than continuous or programmatic tuning.

The widest tuning ranges are achieved in the digital frameworks adopted by Zeinoun et al. [17] and Garcia et al. [18]. The former utilizes an STM32-F446RE [46] MCU, where the PWM signal is software-defined through internal timer registers. This allows for fine-grained, automated frequency control across a broad spectrum of 100–1000 kHz (a 900 kHz span), though it remains subject to the inherent interrupt latencies of a sequential processor [51]. Garcia et al. [18] extend this range further by employing an FPGA-based PWM controller. By utilizing hardware-level logic, this architecture achieves the widest documented tuning range among the ranked systems, facilitating jitter-free frequency sweeps across 303–1315 kHz (a 1012 kHz span) and the execution of complex, high-speed pulsed protocols [52] that are critical for advanced calorimetric analysis.

For technological context, Yöner et al. [20] employ a Wien-bridge oscillator [53] featuring a dual-ganged stereo potentiometer, providing a continuous manual tuning range of 50–200 kHz (a 150 kHz span); this system is excluded from the ranked comparison for the reasons described in “Framework for system-level evaluation of MH architectures" section. While this analog approach offers a wider raw sweep than the fixed or industrial systems evaluated above, the reliance on mechanical tuning lacks the high-resolution logging and programmatic repeatability found in the FPGA- and micro-controller unit (MCU)-driven platforms.

### Amplifier

The selection of the amplifier stage defines the harmonic fidelity and efficiency of the RF signal, with the five ranked studies demonstrating a clear transition from high-power switching topologies to higher-fidelity architectures. At the lowest tier, Chatterjee and Pal [15] utilize an IRF540 MOSFET-based half-bridge Mazzilli inverter. In this configuration, the oscillator and amplifier stages are functionally merged into a single self-resonant unit. While this approach is highly efficient for raw power transfer, it offers the lowest harmonic fidelity among the ranked systems because the output is a distorted quasi-sine wave heavily influenced by the LC tank dynamics, providing no means to suppress parasitic harmonics [54].

A modest improvement in harmonic fidelity is achieved by Attaluri et al. [19], who rely on the AMF LifeSystems 120 kW industrial induction system. At this extreme power scale, harmonic fidelity is secondary to thermal management and switching efficiency. These systems typically produce dirty harmonic profiles as a byproduct of high-power square-wave switching, relying on the high inductance of large-scale coils to provide crude low-pass filtering [55]; unlike Chatterjee and Pal’s system, however, the amplifier and oscillator stages remain functionally distinct, providing marginally greater control over the switching waveform.

Shifting toward modular power switching, Valadez et al. [16] employ a full-H bridge based on the FF150R12RT4 [44] IGBT module. Although this setup provides significant current-handling capability, IGBTs are characterized by slower switching speeds and tail currents during turn-off, which introduce noticeable switching noise and harmonic distortion compared to modern field-effect transistor (FET)-based alternatives [56].

The highest harmonic fidelity among the ranked systems is achieved in the architectures of Zeinoun et al. [17] and Garcia et al. [18], both of which utilize full-bridge inverters with SiC MOSFETs. SiC technology allows for much higher switching frequencies and sharper transitions than standard silicon components, effectively pushing switching harmonics further away from the fundamental frequency [57]. This creates a cleaner excitation field and simplifies the filtering requirements, representing the high-performance ceiling among the ranked designs.

For technological context, Yöner et al. [20] adopt a Class AB amplifier, an architectural choice associated with the highest tier of harmonic fidelity; this system is excluded from the ranked comparison for the reasons described in “Framework for system-level evaluation of MH architectures" section. Unlike the switching nature of the ranked systems above, this linear architecture operates the power transistors in their active region, ensuring the reproduction of a pure sine wave with minimal spectral distortion [58]. However, it must be noted that this superior fidelity comes at a steep cost in efficiency; the power handling capabilities are significantly lower than switching counterparts, as much of the energy is dissipated as heat within the transistors themselves [34]. In practice, coupling a Wien Bridge oscillator with a Class AB amplifier provides a feasible pathway to high-power operation: the oscillator delivers a stable sinusoidal source, while the amplifier scales the signal to the required drive levels. This arrangement has precedent in RF heating circuits, where careful biasing and thermal management enable sustained output without compromising spectral purity. In our design context, the dual-rail supply described above further supports this linear amplification stage, ensuring symmetric voltage rails and compatibility with continuous MH operation.

The preceding evaluation of amplifier architectures, consistent with the majority of the reviewed literature, has assessed each system by its harmonic fidelity, that is, by how closely its output waveform approximates an undistorted sinusoid. It is worth noting that this framing itself carries an assumption worth stating explicitly: that suppressing harmonic content is the correct optimization target. A separate and growing body of work suggests otherwise, showing that deliberately non-harmonic excitation waveforms can enhance nanoparticle heating efficiency relative to conventional sinusoidal excitation of the same frequency and amplitude, rather than representing distortion to be minimized. Allia, Barrera, and Tiberto [59] demonstrated that appropriately shaped non-harmonic driving fields can increase SLP compared with harmonic excitation, a result the same group subsequently extended to trapezoidal and square-wave driving fields. This indicates that waveform shape constitutes a design axis potentially independent of, and possibly in tension with, the harmonic-fidelity criterion ($R_{\mathrm{amp}}$) used in this review: an amplifier optimized to suppress harmonic content is not necessarily optimized for heating efficiency, if a deliberately engineered non-sinusoidal waveform would outperform it. None of the five ranked systems considered here employs a deliberately non-harmonic excitation strategy, and a systematic evaluation of waveform-engineering approaches alongside conventional harmonic fidelity represents an important direction for future instrumentation-focused review work. This future direction is closely related to the spectral-characterization question raised in “Framework for system-level evaluation of MH architectures" section: a full evaluation of waveform-engineering strategies would require not only measuring harmonic content but doing so with a metric sensitive enough to distinguish waveforms of genuine safety and efficiency relevance, a methodological challenge directly addressed by the harmonic residual index proposed in Yöner et al. [20].

### Impedance matching

The strategic selection of an impedance matching circuit is the primary determinant of coupling efficiency, as it dictates how effectively the RF generator compensates for the substantial inductive reactance of the RF coil. At the lowest tier, Garcia et al. [18] represent the baseline archetype with no impedance matching. Without a matching stage, the high inductive reactance of the coil causes a massive impedance mismatch, resulting in the lowest coupling efficiency among the ranked systems, as most power is reflected back to the amplifier rather than being dissipated in the load [60].

A modest improvement is achieved by Chatterjee and Pal [15], who utilize a parallel resonant capacitor. While this creates a tank circuit that achieves resonance, it presents a high impedance to the source at the resonant frequency. This makes it difficult for a voltage-sourced amplifier to drive the high currents necessary for intense magnetic field generation [61], resulting in moderate coupling efficiency.

A different matching philosophy is adopted by Valadez et al. [16], who employ a dual-primary transformer. Although this approach allows for the adjustment of impedance magnitudes by varying the voltage-to-current ratio, it fails to cancel the reactive phase shift inherently. Furthermore, the transformer itself introduces parasitic core losses and leakage inductance, which typically yields low total coupling efficiency [62].

A more robust cancellation of reactance is observed in the capacitive architecture of Zeinoun et al. [17], who utilize a capacitor bank, likely in a series configuration, providing a low-impedance path by canceling the coil’s inductance ($X_L=X_C$). This dedicated bank allows for coarse tunability to align with specific coil geometries [63].

The highest coupling efficiency among the ranked systems is achieved by Attaluri et al. [19], who utilize the internal capacitor banks of the AMF LifeSystems induction system. These integrated banks employ the same single-reactance-cancellation topology as Zeinoun et al.’s capacitor bank, but are additionally professionally engineered for high-power stability and are water-cooled to minimize internal resistive losses, a level of active thermal management not documented for the other ranked systems; this distinction is treated as the deciding factor between the two capacitor-bank-based designs, ensuring a higher percentage of energy reaches the RF coil.

For technological context, Yöner et al. [20] employ a high-pass L-network, an architectural choice associated with the highest tier of coupling efficiency, as it provides two degrees of freedom: it simultaneously cancels the reactive component of the load and transforms the resistive component to perfectly match the source’s output impedance. This mathematical alignment can ensure the maximum possible power transfer, though it requires precise component selection for the specific operating frequency [35]; this system is excluded from the ranked comparison for the reasons described in “Framework for system-level evaluation of MH architectures" section.

## The RF coil design and thermal management

The RF coil design and thermal management stages represent the interface between the RF generator instrumentation and the MNP sample, dictating the spatial distribution of the electromagnetic field and the thermal stability of the experiment. The process begins with the physical architecture of the RF coil, which is engineered to transform the high-amplitude AC from the RF generator into a concentrated magnetic field. The RF coil’s layout, ranging from basic solenoids to multi-coil arrays, controls the magnetic field’s reach and homogeneity. This consistency is vital to ensure every MNP in the sample is excited by the same field intensity. To preserve the integrity of these measurements, a dedicated thermal management strategy should be employed to mitigate the significant resistive heating inherent in high-frequency RF coils. By using advanced cooling methods, these strategies should decouple the magnetic excitation from parasitic resistive heat. This isolation ensures that any measured temperature rise in the sample is strictly the result of magnetic hysteresis.

The integration of spatial field control and thermal isolation defines the performance archetypes observed in current research (Table 3). Applying the scoring framework to these physical designs reveals that RF coil design and thermal management performance ($C_{\mathrm{ordinal}}$) often clusters. This is particularly evident in the trade-offs made between maximizing the magnetic field gradient and implementing comprehensive thermal regulation strategies. Regarding the spatial accuracy of the RF field, Attaluri et al. [19] achieve the highest geometry score among the ranked systems ($R_{\mathrm{geo}}=5$) by utilizing a Maxwell coil configuration, a precisely spaced, anti-parallel dual-coil geometry deliberately engineered to produce a linear field gradient across the target volume [37], representing the same tier of purpose-built spatial field control as a Helmholtz configuration, though directed toward a gradient rather than a homogeneous field objective. For technological context, Yöner et al. [20] utilize a Helmholtz coil configuration alongside solenoid coils using both regular and Litz wire; unlike standard solenoids, which suffer from field decay at the boundaries, a Helmholtz architecture is associated with the highest central field homogeneity among the coil topologies considered in this review [36], though this system is excluded from the ranked comparison for the reasons described in “Framework for system-level evaluation of MH architectures" section.

Beyond spatial homogeneity, the electromagnetic characteristics of the coil, specifically its inductance, play a critical role in determining the efficiency with which the system converts electrical energy into a magnetic field. In this category, Zeinoun et al. [17] and Garcia et al. [18] demonstrate the highest performance among the ranked systems by utilizing an identical coil design that yields an inductance of 13.09 μH. This specific value represents an optimized balance between generating sufficient magnetic flux and maintaining a manageable reactive load for the switching bridge. Because these two systems share the same top-tier inductance value, they are both assigned a mean-rank score of $R_L=4.5$. This scoring reflects their shared design priority of optimizing the inductive load to maximize power throughput within their respective digital architectures.

Conversely, Attaluri et al. [19] demonstrate the pinnacle of thermal isolation, achieving the highest cooling score among the ranked systems ($R_{\mathrm{cs}}=5$) through a closed-loop circulating water system directed at the RF coil itself. This industrial-grade strategy allows the RF coil to operate at near-ambient temperatures even under extreme power loads, effectively eliminating the risk of resistive heat transfer to the MNP sample. In contrast, the remaining ranked systems prioritize simplicity through convective air cooling of the coil. While functional for low-power applications, the absence of coil-directed active cooling in the architectures of Chatterjee and Pal [15], Valadez et al. [16], Zeinoun et al. [17], and Garcia et al. [18] results in a shared performance tier; note that although Zeinoun et al. and Garcia et al. implement a closed-loop circulating water system, this system thermally isolates the MNP sample from the coil wires rather than actively cooling the coil itself, and is therefore scored within the same baseline tier as the purely air-cooled systems, per the criterion defined in “Framework for system-level evaluation of MH architectures" section. Following the mean-ranking protocol, these four systems are each assigned a shared score of $R_{\mathrm{cs}}=2.5$, highlighting the significant performance gap between ambient dissipation and coil-directed liquid cooling. These variations illustrate a broader design trade-off: while specialized laboratory coils, in general, prioritize field strength and homogeneity for precision research, industrial-scale systems focus on the robust thermal management necessary to sustain high-intensity fields over extended durations.

**Table 3:** Functional design choices by each significant study group for the MH RF coil design and thermal management

Study groups$^{C_{\mathrm{ordinal}}}$	Geometry$^{{{R}_\mathrm{geo}}}$	Inductance$^{{R_{L}}}$ (μH)	Cooling strategies$^{{R_{\mathrm{cs}}}}$
Chatterjee and Pal$^{\boldsymbol{1.5}}$ [15]	Two planar coils$^{\boldsymbol{1}}$	1.06 and 1.9$^{\boldsymbol{3}}$	Convective air cooling$^{\boldsymbol{2.5}}$
Valadez et al.$^{\boldsymbol{1.5}}$ [16]	One ellipsoidal planar coil$^{\boldsymbol{2}}$	1.2$^{\boldsymbol{2}}$	Convective air cooling$^{\boldsymbol{2.5}}$
Zeinoun et al.$^{\boldsymbol{3.5}}$ [17]	One solenoid coil with Litz wire$^{\boldsymbol{3.5}}$	13.09$^{\boldsymbol{4.5}}$	Convective air cooling$^{\boldsymbol{2.5}}$
Garcia et al.$^{\boldsymbol{3.5}}$ [18]	One solenoid coil with Litz wire$^{\boldsymbol{3.5}}$	13.09$^{\boldsymbol{4.5}}$	Convective air cooling$^{\boldsymbol{2.5}}$
Attaluri et al.$^{\boldsymbol{5}}$ [19]	One Maxwell coil$^{\boldsymbol{5}}$	0.12$^{\boldsymbol{1}}$	A closed-loop circulating water system$^{\boldsymbol{5}}$
Yöner et al. [20]	Two solenoid coils, (One with regular, one with Litz wire), one Helmholtz coil	6.56, 5.75, and 10.51	Convective air cooling

Bolded superscript numbers (1–5) indicate ordinal ranks assigned to each component for systematic evaluation among the five scored systems, with 5 representing the highest performance and 1 the lowest. Bolded superscript numbers in the first column correspond to the overall coil performance metric $C_{\mathrm{ordinal}}$ for each system. Yöner et al. [20] is presented for technological context but is excluded from ranking to avoid a conflict of interest

Component descriptions and underlying numerical values (e.g., inductance) are as reported in the cited source publications. Ranks for $R_L$ are computed directly from these reported values, whereas ranks for $R_{\mathrm{geo}}$ and $R_{\mathrm{cs}}$ are inferred by the authors of this review by applying the criteria defined in “Framework for system-level evaluation of MH architectures" section

### Geometry

The geometry of the RF coil is a primary determinant of field homogeneity and spatial accuracy within the experimental volume. The reviewed studies demonstrate a variety of geometric configurations all relying on copper wires, ranging from simple planar designs to sophisticated multi-coil arrays. Chatterjee and Pal [15] utilize two planar coils of differing dimensions; the first features 4 turns with an outer diameter of 28.11 mm and an inner diameter of 12.57 mm, while the second incorporates 8 turns with a larger outer diameter of 46.78 mm and the same 12.57 mm inner diameter. While planar coils provide high accessibility for thin-film samples, they exhibit high field gradients along the axial direction [64]. Similarly, Valadez et al. [16] employ a single ellipsoidal planar coil characterized by a 40 × 10 mm inner aperture, constructed using three layers of 4.76 mm diameter copper pipe with each layer consisting of five turns. This ellipsoidal geometry facilitates specific sample orientations but suffers from even more pronounced homogeneity limitations than standard circular planar architectures due to the asymmetric distribution of current density across its primary axes [65].

Zeinoun et al. [17] and Garcia et al. [18] implement an identical solenoid coil architecture consisting of 14 turns with a 58 mm diameter, employing a 30-strand Litz wire where each strand has a diameter of 0.55 mm. Solenoids offer significantly improved field uniformity in the central core compared to planar designs; however, they still suffer from field decay at the coil boundaries [66]. Attaluri et al. [19] utilize a Maxwell coil configuration, engineered via precise anti-parallel dual-coil spacing to produce a linear field gradient across the target volume [37]. Despite the lack of specific dimensional and winding details in the reviewed literature, this configuration’s reliance on the same class of precisely characterized, purpose-engineered dual-coil geometry as the Helmholtz configuration, directed toward a gradient rather than a homogeneity objective results in the highest-tier geometry score among the ranked systems under the field-engineering criterion adopted in this review (“Framework for system-level evaluation of MH architectures" section).

For technological context, Yöner et al. [20] incorporate a three-coil array consisting of two solenoids, one wound with regular wire and one with Litz wire, alongside a Helmholtz coil configuration; this system is excluded from the ranked comparison for the reasons described in “Framework for system-level evaluation of MH architectures" section. All coils in this setup feature a 44 mm diameter, with the Litz wire variant utilizing 7 strands of 0.45 mm diameter. The Helmholtz architecture specifically features 10 turns on each side, a configuration associated with the highest central field homogeneity among the coil topologies considered in this review [36]. The divergence in these geometries reflects a fundamental design trade-off where planar and ellipsoidal coils prioritize accessibility for specific samples at the cost of field consistency, whereas the solenoid and, in particular, Maxwell and Helmholtz configurations prioritize the production of a highly stable magnetic field. It should be emphasized that this ranking reflects the deliberate spatial field-engineering criterion adopted in “Framework for system-level evaluation of MH architectures" section, not a general claim that gradient fields are preferable to homogeneous fields, or vice verse. The Maxwell coil’s high $R_{\mathrm{geo}}$ score reflects the sophistication and precision of its purpose-built gradient design [37], not field homogeneity; a Maxwell geometry is best suited to applications requiring a controlled spatial gradient, such as localization or FFR approaches (“Building the next generation of MH hardware" section), whereas a Helmholtz configuration, as employed for technological context by Yöner et al. [20] remains the appropriate choice where maximal spatial homogeneity is the therapeutic objective. The optimal coil geometry therefore remains application-dependent, and a high $R_{\mathrm{geo}}$ score under this framework should not be read as an indication of universal geometric superiority.

### Inductance

Inductance serves as a critical design parameter in MH systems, as it directly dictates the electromagnetic energy storage capacity of the RF coil. While the Quality factor (*Q*) would ideally provide a more comprehensive measure of coil efficiency and field strength potential [67], the reviewed literature rarely provides the coil wire AC resistance necessary for such a calculation. Consequently, inductance (*L*) was utilized as a functional proxy for coil electromagnetic architecture rather than as a direct performance indicator (see “Framework for system-level evaluation of MH architectures" section). Because the magnetic field inside a coil depends primarily on drive current, turn count, and geometry rather than on inductance itself, inductance is better understood here as an indicator of reactive design burden than of field-generation capacity: at a fixed operating frequency, a higher-inductance coil requires a proportionally higher drive voltage to sustain a given current, and therefore a given field, placing greater demands on the generator and impedance-matching stages. A low-inductance architecture, conversely, permits higher-current, lower-voltage operation, typically at the cost of higher current density and associated resistive losses. The reviewed studies exhibit a wide range of inductance values, reflecting these divergent design philosophies rather than a simple hierarchy of performance. The reviewed studies exhibit a wide range of inductance values, reflecting diverse approaches to coil winding and wire type selection. Within these strategies, one approach is to utilize a coil geometry resulting in low inductance, which is often accompanied by lower coil wire AC resistance. This configuration is a deliberate engineering choice when achieving high magnetic field intensities is prioritized through high-current operation, leveraging low wire resistance to minimize thermal losses rather than relying on high-voltage/low-current driving strategies. Choosing between these two design philosophies is mainly dictated by the RF generator instrumentation’s power handling capabilities. A generator designed for high-current output is better suited for low-inductance coils, whereas a system capable of handling high-voltage swings can effectively drive higher-inductance loads to achieve the necessary field strengths [38].

Chatterjee and Pal [15] reported values of 1.06 and 1.9 μH for their two planar coils, while Valadez et al. [16] employed a single coil with an inductance of 1.2 μH. These values are characteristic of planar and low-turn architectures where specific sample accessibility or high-current driving strategies are prioritized over high-inductance field generation. The mid-to-high range of inductance is represented by the solenoid and multi-coil configurations. Zeinoun et al. [17] and Garcia et al. [18] both utilized the same solenoid coil measured at 13.09 μH, representing the highest inductance reported among the ranked systems. Attaluri et al. [19] utilized a configuration with the lowest reported inductance at 0.12 μH. Rather than indicating a performance limitation, this low-inductance approach suggests a design optimized for high-current throughput or extremely high-frequency impedance matching, where minimizing reactance is essential for system stability [38, 68]. For technological context, Yöner et al. [20] reported a versatile range across their three-coil array, with values of 5.75, 6.56, and 10.51 μH; this system is excluded from the ranked comparison for the reasons described in “Framework for system-level evaluation of MH architectures" section. This variation illustrates how inductance can be tuned across a multi-coil array depending on the specific wire type and geometry used, a design consideration relevant to the broader inductance trade-offs discussed above.

### Cooling strategies

This section addresses thermal management of the RF coil hardware specifically; closed-loop regulation of the therapeutic target temperature, a related but physically distinct problem, is addressed separately in “Auxiliary hardware and multimodal integration" section. The thermal management of the RF coil is a critical, yet frequently overlooked, design parameter that directly impacts system stability and experiment longevity. During RF operation, the skin effect and proximity effect significantly increase the AC resistance of the conductor, leading to substantial resistive heating. This creates a challenging thermal feedback loop: as the temperature rises, the resistivity of the copper increases, further elevating the AC resistance. This increase in AC resistance leads to a direct reduction in the current flowing through the coil for a given input voltage. Since the magnetic field intensity is proportional to the current, this thermal drift results in a weakening magnetic field over time, compromising the consistency of the experimental data. Furthermore, if this heat is not dissipated, the resulting temperature rise can cause a shift in the coil’s inductance, leading to frequency detuning and, in extreme cases, structural failure of the coil insulation or the supporting housing [69].

Despite these risks, several of the ranked studies, namely Chatterjee and Pal [15] and Valadez et al. [16], relied exclusively on convective air cooling. In these configurations, there is no active thermal management system; the coil’s thermal state is governed solely by the efficiency of natural or forced ambient air. This reliance on passive dissipation introduces an uncontrolled variable into the system, which can lead to significant drift in magnetic field intensity during long-duration experiments [70]. Interestingly, both Zeinoun et al. [17] and Garcia et al. [18] implement a closed-loop circulating water system; however, this is not for cooling the RF coil itself, but rather for thermally isolating the MNPs from the coil wires. Because this cooling strategy does not directly target the coil, these two systems are scored within the same baseline cooling tier as the purely air-cooled designs, per the criterion defined in “Framework for system-level evaluation of MH architectures" section, yielding a shared score of $R_{\mathrm{cs}}=2.5$ across all four systems. This specific architectural choice indicates that the RF coil wires heat considerably, necessitating a dedicated barrier to prevent conduction from interfering with sample temperature readings. For researchers aiming for high-current applications or extended duty cycles, the lack of coil-directed active cooling in these designs represents a significant design weakness, as the coil temperature becomes the primary limiting factor for the maximum achievable SLP.

In contrast, Attaluri et al. [19] implemented a closed-loop circulating water system directed at the coil itself, achieving the highest cooling score among the ranked systems ($R_{\mathrm{cs}}=5$) and representing the gold standard for RF heating applications. By utilizing copper tubing or specialized cooling jackets to circulate water directly through or around the coil, heat is removed far more efficiently than is possible with air. This active management allows for precise thermal equilibrium, maintaining a constant coil temperature to prevent inductance drift and ensuring repeatable experimental conditions [70]. Furthermore, it enables high power density by allowing the use of significantly higher currents, and thus higher magnetic field strengths without the risk of overheating the conductor. Crucially, active cooling provides better MNP sample protection by minimizing the radiative heat transfer from a hot coil to the sample. This is essential to ensure that any observed temperature rise in the MNP solution is due to magnetic loss rather than parasitic heating from the coil wires itself [71]. For technological context, Yöner et al. [20] likewise relied on convective air cooling without coil-directed active thermal management, though this system is excluded from the ranked comparison for the reasons described in “Framework for system-level evaluation of MH architectures" section. The transition from passive convective cooling to active liquid cooling is often the primary differentiator between bench-top prototypes and robust, clinical-grade MH systems.

## Auxiliary hardware and multimodal integration

While the fundamental goal of MH is the controlled elevation of temperature, the clinical efficacy of the hardware depends largely on the sophistication of its auxiliary modules, which range from automated thermal regulation to high-resolution mechanical tracking. In the system developed by Chatterjee and Pal [15], the integration of a PI controller serves as the primary auxiliary module for maintaining precise thermal regulation during MH therapy. Unlike open-loop systems that simply apply a constant field, this architecture utilizes a closed-loop feedback mechanism to ensure that the target temperature is reached rapidly and maintained without significant fluctuation. The control loop begins with a M/s Optris [72] brand infrared (IR) radiation thermometer (8–14 μm spectral range), which acts as the non-contact sensing interface. By reading the thermal radiation emitted from the sample, the IR thermometer provides real-time temperature data without the risk of electromagnetic interference that traditional metallic thermocouples would face within the RF field [73]. This temperature reading is continuously fed into the PI controller, which calculates the error between the current sample temperature and the desired set-point. The PI controller then generates a proportional analog output signal to modulate the power delivered by the RF generator instrumentation to the RF coil. By adjusting the current flow in response to this analog signal, the system can dampen the heating rate as the target temperature is approached, effectively preventing overshoot and ensuring a stable plateau. This automated tuning of the MH therapy allows for consistent experimental conditions, which is crucial for accurately determining the SLP and ensuring the safety of biological tissues during the heating process.

Whereas the previous architecture optimizes the heating process through surface-level thermal feedback, other systems, such as the one developed by Valadez et al. [16], shift the focus toward the internal mechanical environment by integrating auxiliary imaging modalities to resolve the spatial distribution of the thermal dose. The development of the magneto-motive ultrasound (MMUS) auxiliary module represents a critical technical advancement within the theranostic system introduced by Valadez et al. [16], specifically designed to bridge the gap between high-power RF therapy and high-sensitivity diagnostics. While standard ultrasound provides structural imaging, it often lacks the inherent contrast required to distinguish MNPs from surrounding dense tissue [74]. The MMUS module addresses this limitation by utilizing a specialized high-capacity capacitor discharge topology engineered to deliver short-duration, high-intensity magnetic pulses. This subsystem is capable of generating peak currents up to 2.16 kA, which in turn produces a robust magnetic field gradient of approximately 205 kA/m. These transient pulses exert a magneto-motive force, essentially a pulling action on the MNPs, inducing micrometer-scale tissue displacements, which are captured using a Verasonics Vantage 128 [75] ultrasound platform, which is synchronized with the magnetic pulses to map MNP distribution with high precision.

The successful integration of this MMUS tracking with MH requires a sophisticated architectural approach to resolve the inherent frequency and power incompatibilities between the two modalities. In the system described by Valadez et al. [16], a shared-coil strategy is employed where a single ellipsoidal-planar coil serves as both the heating element for therapy and the displacement inductor for imaging. To prevent electronic interference and hardware damage, a high-frequency ferrite transformer is utilized as a coupling mechanism, effectively isolating the continuous-wave MH generator from the high-voltage MMUS discharge circuit. This integration is managed through an interleaved timing protocol orchestrated by an STM32-based [46] MCU, which briefly pauses the hyperthermia wave to deliver the diagnostic MMUS pulse, ensuring a seamless transition between treatment and monitoring.

Beyond simple localization, this multimodal integration allows for the observation of dynamic changes in tissue biomechanics during the heating process. As the MH module raises the temperature of the target area, the MMUS module serves as a real-time diagnostic tool to assess the resulting softening effect in the tissue matrix. Experimental data from the study indicate that as temperature increases, the magnitude of the magneto-motive displacement often shifts, reflecting changes in the elasticity of the environment surrounding the MNPs. This synergistic relationship effectively transforms the device into a comprehensive theranostic platform, where the auxiliary MMUS tracking module provides the critical feedback loop necessary to quantify MNP concentration, verify targeting accuracy, and monitor the thermal dose delivered to the site.

## Building the next generation of MH hardware

**Fig. 3:**
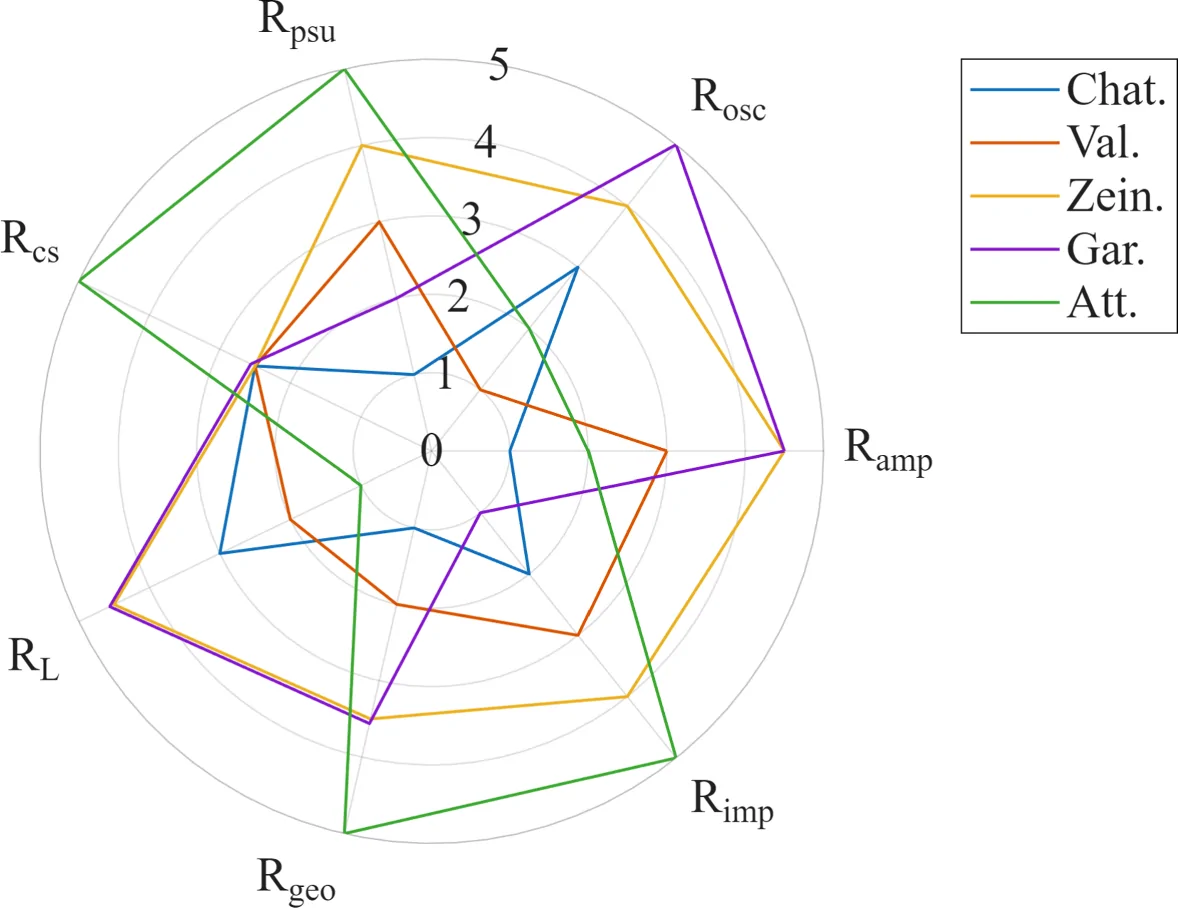
Comparative hardware profile of five prominent MH designs across seven engineering dimensions: DC power supply (*R*_psu_), oscillator (*R*_osc_), amplifier (*R*_amp_), impedance matching (*R*_imp_), coil geometry (*R*_geo_), inductance (*R*_L_), and cooling strategy (*R*_cs_)

Advancing the field requires a cold-eyed retrospective assessment; the performance profiles synthesized in the comparative spider charts reveal an inconsistent landscape that defines the current state of the art. The first spider chart (Fig. 3), detailing the hardware profile across seven dimensions: DC power supply ($R_{\mathrm{psu}}$), oscillator ($R_{\mathrm{osc}}$), amplifier ($R_{\mathrm{amp}}$), impedance matching ($R_{\mathrm{imp}}$), coil geometry ($R_{\mathrm{geo}}$), inductance ($R_{L}$), and cooling strategies ($R_{\mathrm{cs}}$) exposes a systemic vulnerability: cooling strategy scores are critically limited across nearly all designs, with Attaluri et al. [19] being a notable exception. Beyond this shared weakness, the scoring across different MH design stages is remarkably fragmented. There are no cohesive design trends or universal prioritization; instead, each research group appears to have prioritized disparate aspects of the system. While these could be framed as deliberate design sacrifices, this lack of uniformity actually underscores a deeper issue: the absence of established engineering standards for RF generator instrumentation, coil design, and thermal management within the MH field.

Coil geometry represents a critical design trade-off. Planar coils offer strong field confinement for shallow, targeted therapy, but their penetration depth is limited [76]. Solenoid coils enable deeper penetration suitable for torso or limb heating, though at the expense of spatial selectivity [77]. Helmholtz configurations provide highly uniform fields across larger volumes, making them attractive for systemic or whole-region heating [78]. These geometries thus embody different balances between depth, uniformity, and confinement, underscoring the need for application-specific coil design rather than a universal solution.

The instrumentation-focused comparison developed in this review is complemented by a distinct and growing body of work that directly links specific coil-design choices to measured nanoparticle heating outcomes under matched experimental conditions, an approach the confounding factors discussed in “Framework for system-level evaluation of MH architectures" section make difficult to incorporate into a cross-system ordinal ranking, but which is nonetheless directly relevant to the field’s broader trajectory toward evaluating instrumentation by delivered thermal effect rather than by architecture alone. Choi et al. [14], cited earlier in this review, provide a representative example: a series-resonated Helmholtz coil system is designed, and its coil geometry, driver circuit, and resonance strategy are directly evaluated against a measured SAR of 6.27 W/g under defined field and nanoparticle conditions, with the resulting heating performance further validated in vivo. Similarly, Brizi et al. [79] designed a coil to focus the magnetic field at the frequency maximizing heat release for a chosen nanoparticle formulation, and validated this focusing effect experimentally across multiple nanoparticle samples. Such single-system studies, in which coil design and measured heating outcome are reported together under controlled, matched conditions, represent a necessary methodological complement to a cross-system architectural comparison such as the one presented here: they establish the causal, hardware-to-outcome link that a heterogeneous, literature-derived comparison across independently developed systems, evaluated under non-matched nanoparticle and field conditions, cannot establish. We view systematic aggregation of this class of matched-condition, instrumentation-to-outcome studies, potentially extending to a comparative framework in its own right, as an important direction for future review work bridging the instrumentation-focused perspective of the present review with the nanoparticle-heating-effectiveness literature.

The scope of this review, in common with the majority of the reviewed literature, has focused on instrumentation generating conventional, linearly oscillating AMFs. A growing body of work, however, indicates that the vector nature of the applied field, not only its amplitude and frequency, may materially influence nanoparticle heating behavior, and that this represents a meaningful extension of the instrumentation design space rather than a minor variant of AMF excitation.

Skumiel and Wojciechowski [80] demonstrated a two-phase RMF generation system operating from 38–190 kHz at field amplitudes up to 20 kA/m, showing that the heating-rate dependence on field amplitude (*dT*/*dt*   $H^{2}$), well established for AMF systems, breaks down at higher RMF amplitudes, indicating pronounced nonlinear behavior not captured by amplitude alone. Wojciechowski et al. [81] subsequently presented a fully designed, constructed, and experimentally validated RMF generation system incorporating three-dimensional finite-element magnetic-circuit modeling, demonstrating that RMF generator design can integrate electromagnetic modeling, magnetic-circuit optimization, and turn-count design in a manner directly analogous to the AMF generator-design considerations discussed in “RF generator instrumentation" section. Skumiel [82] further compared two-phase and three-phase RMF architectures, finding that a three-phase system achieves approximately twice the field amplitude of a two-phase system at the same supply voltage, at the cost of correspondingly higher power consumption, an instrumentation-level trade-off directly analogous to the power-versus-complexity trade-offs identified for AMF generator architectures in “DC power supply" section.

Beyond instrumentation design, RMF excitation may also alter the underlying heat-generation mechanism itself. Barrera et al. [59] modeled the magnetic response of nanoparticles under a uniformly rotating field and showed, via a coupled thermal model, that RMF excitation can permit more than a twofold reduction in the nanoparticle dose required to reach a target tissue temperature compared with a linear field of equivalent amplitude. This result is notable in the present context because it indicates that instrumentation-level metrics such as field amplitude and generator architecture, as evaluated throughout this review, may be insufficient on their own to characterize therapeutic effectiveness without also specifying the field’s vector configuration.

This finding should not, however, be read as establishing a general superiority of rotating over oscillating fields. Theoretical work by Iszály et al. [83], addressing superlocalized hyperthermia combining a driving field with a static field gradient, found the converse result in the low-frequency regime relevant to clinical hyperthermia: an oscillating field yielded approximately twice the ILP and SAR of a rotating field with equivalent spatial localization ability. Taken together, this literature indicates that the relative heating efficiency of rotating versus oscillating fields is regime- and application-dependent, governed by factors including field amplitude, frequency, nanoparticle anisotropy, and whether spatial localization is a simultaneous design objective, rather than a settled, one-directional result.

Given the distinct multi-phase generator architectures and magnetic-circuit topologies RMF systems require, a rigorous comparative evaluation of RMF instrumentation using the ordinal framework developed in “Framework for system-level evaluation of MH architectures" section was judged to be outside the scope of the present review. We identify a dedicated engineering-focused review comparing RMF and AMF instrumentation architectures under a unified evaluation framework as an important and currently unaddressed direction for future work, and note that any such framework would need to account for the amplitude-, frequency-, and application-dependence of the relative heating efficiency of the two field configurations, rather than treating one as universally preferable.

**Fig. 4:**
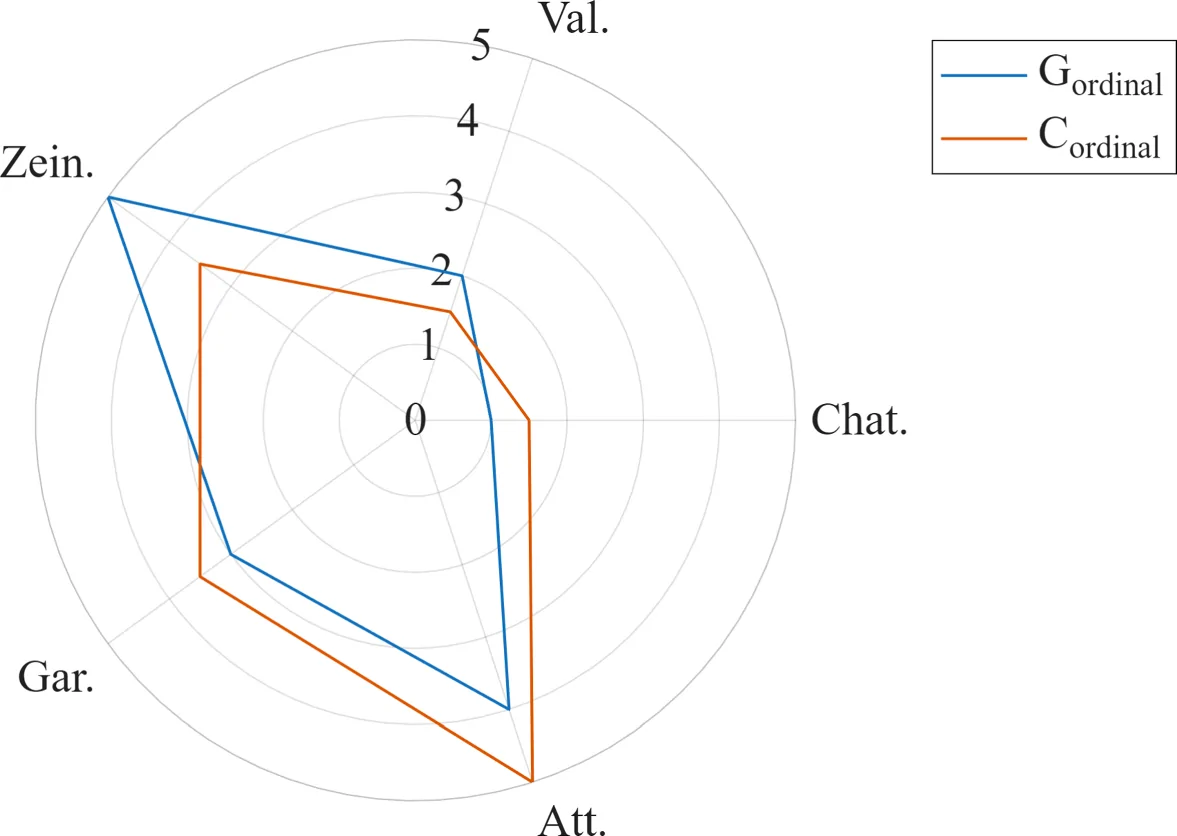
Global performance landscape of the reviewed MH systems, comparing the overall performance of the RF generator instrumentation ($G_{\mathrm{ordinal}}$) with the overall performance of the RF coil design and thermal management ($C_{\mathrm{ordinal}}$) across all five design archetypes

The global performance landscape (Fig. 4), which contrasts overall RF generator instrumentation ($G_{\mathrm{ordinal}}$) with coil design and thermal management ($C_{\mathrm{ordinal}}$), further clarifies this divide. Our engineering-focused scoring reveals that Zeinoun et al. [17] and Garcia et al. [18] demonstrate the strongest joint performance across both domains among the ranked systems, reflecting their shared prioritization of digitally controlled, SiC-based RF generation and high-inductance solenoid coil design. In contrast, Chatterjee and Pal [15] and Valadez et al. [16] occupy the lower end of both metrics, reflecting simpler, lower-cost architectures that prioritize accessibility and rapid prototyping over comprehensive engineering optimization. Attaluri et al. [19] presents a distinct profile, combining a mid-range $G_{\mathrm{ordinal}}$ with the highest $C_{\mathrm{ordinal}}$ among the ranked systems, driven almost entirely by coil-directed active cooling and Maxwell-coil geometry rather than by generator-side sophistication. For additional technological context, Yöner et al. [20], although excluded from the ranked comparison for the reasons described in Sect. Framework for system-level evaluation of MH architectures, exhibits architectural choices, such as a Class AB amplifier and a Helmholtz coil configuration, that would be associated with high harmonic fidelity and geometric homogeneity under the criteria defined in this review, though this system’s single-source configuration also results in comparatively limited MNP heating capacity relative to the multi-source or industrial-scale ranked systems. These five ranked investigations thus represent a critical crossroads, proving that without a unified optimization framework, the field may continue to oscillate between high-power experimental tools and well-engineered but output-limited prototypes.

To move beyond these fragmented design architectures, future engineering efforts must focus on unifying high-performance output with rigorous instrumentation standards. The path forward demands a transition from static, purpose-built experimental setups toward frequency-agile and thermally autonomous platforms. This evolution is supported by five engineering priorities. The first three follow directly from the comparative scoring presented in this review; the remaining two represent broader engineering directions relevant to the field that are not directly evidenced by the reviewed systems, but are included to situate near-term hardware priorities within the longer-term trajectory of MH instrumentation. First, the transition toward dynamic spectral agility is paramount. As evidenced by the disparate scoring in $R_{\mathrm{osc}}$ and $R_{\mathrm{imp}}$, current high-power systems are often restricted to narrow operational bandwidths, frequently centered around a fixed frequency to protect internal matching circuitry. This limitation forces researchers to adapt their MNPs to the hardware rather than the reverse. Future designs must incorporate automated, real-time impedance matching to allow for broad spectral sweeps, enabling both the characterization of MNP response across a wide range of frequencies without manual hardware reconfiguration, and system compatibility with various MNPs. At the same time, spectral agility must be balanced against established safety constraints: in practical hyperthermia therapy, varying coil current rather than frequency is generally preferable, as the product H×f is limited by the Atkinson–Brezovich [31, 32] or Hergt–Dutz criterion [33]. Increasing frequency beyond a few kHz exacerbates eddy current heating in non-target tissues [84, 85]. This eddy-current-driven, non-specific heating exemplifies the electromagnetic-exposure/thermal-safety distinction introduced in “Introduction" section: the f×H product constrains electromagnetic exposure in aggregate, but the resulting thermal-safety outcome, whether a given exposure produces a clinically significant local hot spot, additionally depends on patient-specific body geometry, tissue conductivity, and the spatial distribution of the induced field, factors the f×H criterion alone does not capture. Consequently, future MH platforms should emphasize current modulation to achieve therapeutic field variation while reserving frequency agility for characterization studies rather than continuous clinical operation.

This spectral-agility priority should be paired with standardized, quantitative reporting of amplifier output quality. As discussed in “Framework for system-level evaluation of MH architectures" section, the present review was constrained to an architectural-tier characterization of harmonic fidelity because THD, individual harmonic amplitudes, and frequency/amplitude stability are inconsistently reported across the surveyed systems; even where THD is reported, it can obscure safety-relevant spectral detail that a more discriminating metric, such as the harmonic residual index proposed in Yöner et al. [20], is designed to capture. Consistent adoption of such standardized spectral-purity metrics across future MH instrumentation reports would allow subsequent comparative reviews to rank amplifier performance directly, rather than relying on the architectural-tier proxy necessitated here.

Second, the field must adopt integrated active thermal management as a core design requirement rather than an auxiliary module. The systemic failure in ${R}_{\mathrm{cs}}$ scores across the reviewed literature indicates that thermal drift remains a major uncontrolled variable in MH experiments. Building upon the water-cooling isolation strategies seen in Garcia et al. [18] and Zeinoun et al. [17], the next generation of hardware must implement active cooling directly within the RF coil architecture. Drawing a parallel to the sophisticated cooling paradigms established in Magnetic Resonance Imaging (MRI) systems, it is suggested that future MH platforms could significantly benefit from transitioning toward integrated liquid-cooled manifolds, such as high-flow gradient cooling or hollow-core conductors [86]. This transition is particularly critical when considering the eventual clinical scaling of MH technology. While current experimental devices typically drive small-scale coils for small animal models or in vitro samples, a successful clinical transition will necessitate human-scale coils capable of encompassing the torso or limbs. As the geometry scales, the increased inductive load and the power required to maintain therapeutic field intensities across larger volumes will result in substantially higher resistive losses and heat generation [87]. Sophisticated, high-efficiency cooling is therefore not merely a technical luxury, but a fundamental requirement to manage the thermal footprint of larger systems, ensuring that the electromagnetic field remains decoupled from parasitic thermal output during human-scale interventions.

Third, building on the auxiliary modalities discussed above, the integration of autonomous, multimodal feedback loops represents a natural extension of the systems reviewed here. While Valadez et al. [16] demonstrated the utility of MMUS tracking, and Chatterjee and Pal [15] introduced foundational PI-control logic, these modalities often remain siloed. Future hardware should integrate these modalities into a closed-loop system where real-time diagnostic data such as temperature and MNP localization directly modulate the RF generator’s frequency and power. This would transform MH devices from passive energy sources into intelligent therapeutic platforms capable of navigating the complex, shifting dynamics of tissue–MNP interactions autonomously.

Fourth, beyond hardware-level control, the integration of Artificial Intelligence (AI) represents a longer-term direction for bridging predictable in vitro heating behavior with the highly non-linear, patient-specific thermoregulatory response observed clinically, where perfusion changes act as a biological cooling mechanism that varies with tumor type, location, and patient physiology [88–90]. Because such multi-dimensional, time-varying thermal dynamics are difficult to capture with classical models or fixed PI-control loops, future MH hardware may benefit from pairing with machine learning algorithms capable of processing multimodal sensor data to predict and counteract these effects in real time. This direction is not evidenced by the systems reviewed here, none of which incorporate AI-based control, but is noted as a longer-term complement to the hardware-level agility and thermal management priorities identified above.

Finally, spatial restriction of magnetic energy is a separate engineering direction addressing thermal safety directly, rather than electromagnetic exposure alone: it is relevant to preventing non-specific eddy current heating in tumor-free regions as RF power increases [91, 92]. One approach under investigation combines the primary RF coil with high-precision DC gradient coils to generate a FFR, in which a superimposed static gradient quenches MNP heating everywhere except at the gradient’s zero-point [93, 94]; current implementations, however, remain limited by static coil geometries. As with AI-based control, this direction is not represented among the reviewed systems, none of which implement FFR or DC gradient architectures, but is included here as a complementary avenue for future spatial-confinement research.

## Conclusion

This work is presented as a critical review of engineering trade-offs in the design of contemporary MH instrumentation, rather than as a definitive ranking of the best available solutions; the ordinal comparisons developed here are intended to structure and make explicit these trade-offs, not to identify a single optimal system. This review underscores that advancing MH toward more standardized, reproducible instrumentation is fundamentally tethered to the evolution of its underlying hardware. We note that the scope of this review is deliberately bounded to the instrumentation chain terminating at magnetic-field generation; extending system-level optimization to encompass the full physiological chain, magnetic nanoparticle response, heat generation and transfer, tissue temperature, thermal dose, and biological effect represents an important and complementary direction for future engineering-oriented review work, contingent on more consistent reporting of SLP/SAR/ILP and field-dosimetry data across the instrumentation literature. While material science has advanced rapidly, the field currently lacks a unified engineering standard for RF generator instrumentation, RF coil design and thermal management, leading to significant trade-offs between raw power and signal fidelity. The widespread deficiency in active thermal management and real-time impedance matching remains a critical vulnerability, as uncontrolled thermal drift and static configurations compromise experimental reproducibility. To overcome these technical bottlenecks, next-generation hardware must prioritize a hardware-first optimization strategy, integrating active liquid-cooled manifolds, autonomous feedback loops for dose regulation, and advanced spatial steering to transform MH instrumentation into a more standardized, high-precision engineering platform for future therapeutic development.

## Data Availability

No datasets were generated or analyzed during the current study.

## References

[1] Szwed M, Marczak A. Application of nanoparticles for magnetic hyperthermia for cancer treatment the current state of knowledge. Cancers. 2024;16(6):1156. 10.3390/cancers16061156. PMC10969623 38539491

[2] Gilchrist RK, Medal R, Shorey WD, Hanselman RC, Parrott JC, Taylor CB. Selective inductive heating of lymph nodes. Ann Surg. 1957;146(4):596–606. 10.1097/00000658-195710000-00007. PMC1450524 13470751

[3] Fiorentini G, Sarti D, Mambrini A, Mattioli G, Bonucci M, Ginocchi L, et al. Locoregional hyperthermia in cancer treatment: a narrative review with updates and perspectives. Onco. 2025;5(2):26. 10.3390/onco5020026.

[4] Tishin AM. Modern applications of magnetic materials: trends and challenges. Magnet Magn Mater Spintr. 2025;126:1571–89. 10.1134/S0031918X25601908.

[5] Shakeri-Zadeh A, Bulte JWM. Imaging-guided precision hyperthermia with magnetic nanoparticles. Nat Rev Bioeng. 2025;3:245–60. 10.1038/s44222-024-00257-3. PMC12011369 40260131

[6] Peiravi M, Eslami H, Ansari M, Zare-Zardini H. Magnetic hyperthermia: potentials and limitations. J Indian Chem Soc. 2022;99(1):100269. 10.1016/j.jics.2021.100269.

[7] Vaupel P, Piazena H, Notter M, Thomsen AR, Grosu AL, Scholkmann F, et al. From localized mild hyperthermia to improved tumor oxygenation: physiological mechanisms critically involved in oncologic thermo-radio-immunotherapy. Cancers. 2023;15(5):1394. 10.3390/cancers15051394. PMC10000497 36900190

[8] Shetake NG, Balla MMS, Kumar A, Pandey BN. Magnetic hyperthermia therapy an emerging modality of cancer treatment in combination with radiotherapy. J Radiat Cancer Res. 2016;7(1):13–7. 10.4103/0973-0168.184606.

[9] Stigliano RV, Danelyan I, Gabriadze G, Shoshiashvili L, Baker I, Hoopes PJ, et al. Alternating magnetic field guiding system for MNP hyperthermia treatment of deep-seated cancers. Int J Hyperth. 2024;41(1):2391008. 10.1080/02656736.2024.2391008. 39205623

[10] Qian KY, Song Y, Yan X, Dong L, Xue J, Xu Y, et al. Injectable ferrimagnetic silk fibroin hydrogel for magnetic hyperthermia ablation of deep tumor. Biomaterials. 2020;259:120299. 10.1016/j.biomaterials.2020.120299. 32827797

[11] Naik S, Munjal S. Nanotechnology-driven advances in magnetic hyperthermia: a comprehensive review. Biomed Mater Dev. 2025. 10.1007/s44174-025-00593-x.

[12] Nguyen LH, Oanh VTK, Nam PH, Doan DH, Truong NX, Ca NX, et al. Increase of magnetic hyperthermia efficiency due to optimal size of particles: theoretical and experimental results. J Nanopart Res. 2020. 10.1007/s11051-020-04986-5.

[13] Gavilan H, Avugadda SK, Fernandez-Cabada T, Soni N, Cassani M, Mai BT, et al. Magnetic nanoparticles and clusters for magnetic hyperthermia: optimizing their heat performance and developing combinatorial therapies to tackle cancer. Chem Soc Rev. 2021;50:11614–67. 10.1039/D1CS00427A. 34661212

[14] Choi J, Song M, Kim H, Lee H, Kim H, Park S. Design and experimental validation of series-resonated helmholtz coil system for magnetic nanoparticle hyperthermia. IEEE J Electromagn RF Microwav Med Biol. 2026. 10.1109/JERM.2026.3664393.

[15] Chatterjee M, Pal S. Design and performance evaluation of magnetic hyperthermia instrument with embedded pi control. Electromagn Biol Med. 2025;44:1–15. 10.1080/15368378.2025.2524547. 40581938

[16] Valadez EEM, Uliana JH, Vicente TT, Carneiro AAO, Pavan TZ. Fully-automated theranostic system integrating magnetomotive ultrasound and magnetic hyperthermia. IEEE Trans Instrum Meas. 2025;74:1–11. 10.1109/TIM.2025.3542151.

[17] Zeinoun M, Serrano D, Medina PT, Garcia O, Vasic M, Serrani-Olmedo JJ. Configurable high-frequency alternating magnetic field generator for nanomedical magnetic hyperthermia applications. IEEE Access. 2021;9:105805–16. 10.1109/ACCESS.2021.3099428.

[18] Garcia O, Moreno-Arrones N, Cuesta AB, Gutierres A, Alou P, Oliver JA, et al. Development and testing of a new instrument for researching on cancer treatment technologies based on magnetic hyperthermia. IEEE J Emerg Select Top Power Electr. 2016;4(1):243–51. 10.1109/JESTPE.2015.2479092.

[19] Attaluri A, Jackowski J, Sharma A, Kandala SK, Nemkov V, Yakey C, et al. Design and construction of a maxwell-type induction coil for magnetic nanoparticle hyperthermia. Int J Hyperth. 2020;37(1):1–14. 10.1080/02656736.2019.1704448. PMC6956739 31918595

[20] Yöner SI, Ozcan A, Akin O. Characterizing magnetic hyperthermia RF generator spectral purity at 150 kHz: The harmonic residual index as a standardized metric for inductive safety. SSRN Electr J. 2026. 10.2139/ssrn.6735899.

[21] Yoner SI. Design and optimization of a nanoparticle based magnetic hyperthermia device. Istanbul: Bogazici University; 2025.

[22] MagForce Nanotechnologies AG: NanoActivator F100: Clinical Induction Heating System for Magnetic Hyperthermia. MagForce Nanotechnologies AG, Berlin, Germany. 2026. MagForce Nanotechnologies AG. https://www.magforce.de/. Accessed on 17 Aug 2026.

[23] nanoTherics Ltd: magneTherm: High-Frequency Magnetic Field Generator for Nanoparticle Research. nanoTherics Ltd, Warrington, United Kingdom. nanoTherics Ltd. 2026. https://www.nanotherics.com/. Accessed 17 Aug 2026.

[24] Gneveckow U, Jordan A, Scholz R, Brüss V, Waldöfner N, Ricke J, et al. Description and characterization of the novel hyperthermia- and thermoablation-system MFH 300f for clinical magnetic fluid hyperthermia. Med Phys. 2004;31(6):1444–51. 10.1118/1.1748629. 15259647

[25] Stauffer PR, Cetas TC, Jones RC. Magnetic induction heating of ferromagnetic implants for inducing localized hyperthermia in deep-seated tumors. IEEE Trans Biomed Eng. 1984;31(2):235–51. 10.1109/TBME.1984.325772. 6706353

[26] Levi-Polyachenko N, et al. A validated methodological approach to prove the safety of clinical electromagnetic induction systems in magnetic hyperthermia. Cancers. 2024;16(3):621. 10.3390/cancers16030621. PMC10854696 38339373

[27] Sadaphal V, Prasad B, Kay W, Nehring L, Nyugen T, Tepper J, et al. Feasibility of heating metal implants with alternating magnetic fields (AMF) in scaled up models. Int J Hyperth. 2022;39(1):81–96. 10.1080/02656736.2021.2011434. 34949138

[28] Dennis CL, Ivkov R. Physics of heat generation using magnetic nanoparticles for hyperthermia. Int J Hyperth. 2013;29(8):715–29. 10.3109/02656736.2013.836758. 24131317

[29] Verde EL, Landi GT, Gomes JA, Sousa MH, Bakuzis AF. Magnetic hyperthermia investigation of cobalt ferrite nanoparticles: comparison between experiment, linear response theory, and dynamic hysteresis simulations. J Appl Phys. 2012;111:123902. 10.1063/1.4729271.

[30] Carrey J, Mehdaoui B, Respaud M. Simple models for dynamic hysteresis loop calculations of magnetic single-domain nanoparticles: application to magnetic hyperthermia optimization. J Appl Phys. 2011;109(8):083921. 10.1063/1.3551582.

[31] Atkinson WJ, Brezovich IA, Chakraborty DP. Usable frequencies in hyperthermia with thermal seeds. IEEE Transact Biomed Eng BME. 1984;31(1):70–5. 10.1109/TBME.1984.325372. 6724612

[32] Brezovich IA, Atkinson WJ, Lilly MB. Local hyperthermia with interstitial techniques. Can Res. 1984;44(10 Supplement):4752–6. 6380712

[33] Hergt R, Dutz S. Magnetic particle hyperthermia biophysical limitations of a visionary tumour therapy. J Magn Magn Mater. 2007;311(1):187–92. 10.1016/j.jmmm.2006.10.1156.

[34] Self D. Audio Power Amplifier Design Handbook. 4th ed. Burlington: Newnes; 2006.

[35] Kazimierczuk MK. High-frequency magnetic components. 2nd ed. Chichester: Wiley; 2013.

[36] Batista DD, Granziera F, Tosin MC, Melo LF. Three-axial helmholtz coil design and validation for aerospace applications. IEEE Trans Aerosp Electron Syst. 2018;54(1):392–403. 10.1109/TAES.2017.2760560.

[37] Ghosh SK, Thakur V, Chowdhury SR. Design and simulation of helmholtz coil and maxwell coil for low cost and low magnetic field MRI machine. Adv Mater Proc. 2017;2(12):802–6. 10.5185/amp.2017/918.

[38] Erickson RW, Maksimovic D. Fundamentals of Power Electronics. 3rd ed. Cham: Springer; 2020.

[39] Pozar DM. Microwave Engineering. 4th ed. Hoboken: Wiley; 2012.

[40] Yip PCL. High-frequency circuit design and measurements. 1st ed. Boston, MA: Springer; 1990.

[41] Scientific India. https://www.scientificindia.com/. Accessed on 06 May 2026.

[42] Intertechnology V. 2008. IRF540 power MOSFET datasheet. https://www.vishay.com/docs/91021/irf540.pdf. Accessed on 06 May 2026.

[43] Instruments T. 2015. UCC3895DW phase-shift PWM controller datasheet. https://www.ti.com/lit/ds/symlink/ucc3895.pdf. Accessed on 06 May 2026.

[44] AG, I.T.: FF150R12RT4 IGBT module datasheet. 2013. https://www.infineon.com/dgdl/ff150r12rt4.pdf. Accessed on 06 May 2026.

[45] AMF Life. https://amflife.com/. Accessed on 6 May 2026.

[46] STMicroelectronics: STM32F446MC microcontroller datasheet. 2015. https://www.st.com/resource/en/datasheet/stm32f446mc.pdf. Accessed on 6 May 2026.

[47] Mean Well. https://www.meanwell.com/. Accessed on 6 May 2026.

[48] Zhang N, Li D, Zhang V, Chen A. Reduce conducted emi in automotive buck converter applications. Technical Report SNVA886, Texas Instruments. 2019. https://www.ti.com/lit/an/snva886/snva886.pdf.

[49] Pressman AI, Billings K, Morey T. Switching power supply design. 3rd ed. New York: McGraw-Hill; 2009.

[50] Lucia O, Burd JM, Millan I, Acero J, Barragan LA. Efficiency-oriented design of zvs half-bridge series resonant inverter with variable frequency duty cycle control. IEEE Transact Power Electr. 2010;25(7):1671–4. 10.1109/TPEL.2010.2042461.

[51] Noergaard T. Embedded systems architecture: a comprehensive guide for engineers and programmers. 2nd ed. Burlington, MA: Newnes; 2012.

[52] Tu K, Tang X, Yu C, Josipovic L, Chu Z. FPGA EDA. 1st ed. Singapore: Springer; 2024.

[53] Zhang Y. Essential microelectronic circuits. 2nd ed. Bristol: IOP Publishing; 2025.

[54] Andreycak B. Zero voltage switching resonant power conversion. Technical report, Texas Instruments. 2001. https://www.ti.com/lit/ml/slup089/slup089.pdf.

[55] Rudnev V, Loveless D, Cook RL. Handbook of induction heating. 2nd ed. Boca Raton, FL: CRC Press; 2017.

[56] Sheng K, Williams BW, Finney SJ. A review of IGBT models. IEEE Trans Power Electron. 2000;15(6):1250–66. 10.1109/63.892840.

[57] Ahmad SS, Urabinahatti C, Prasad KNV, Narayanan G. High-switching-frequency sic power converter for high-speed switched reluctance machine. IEEE Trans Ind Appl. 2021;57(6):6069–82. 10.1109/TIA.2021.3111540.

[58] Walker JLB. Handbook of RF and microwave power amplifiers. 1st ed. Cambridge: Cambridge University Press; 2011.

[59] Barrera G, Allia P, Tiberto P. Magnetic nanoparticle hyperthermia enhanced by a rotating field. Phys Rev Appl. 2024;21:064037. 10.1103/PhysRevApplied.21.064037.

[60] Traficante DD. Impedance: what it is, and why it must be matched. Concepts Magn Resonance. 1989;1:73–92. 10.1002/cmr.1820010205.

[61] Guittienne P, Howling A, Furno I. Resonant network antennas for radio-frequency plasma sources. 1st ed. Bristol: IOP Publishing; 2024.

[62] Darraji R, Honari MM, Mirzavand R, Ghannouchi FM, Mousavi P. Wideband two-section impedance transformer with flat real-to-real impedance matching. IEEE Microwave Wirel Compon Lett. 2016;26(5):313–5. 10.1109/LMWC.2016.2548997.

[63] Pang H, Xu F, Liu W, Tse CK, Chau KT. Impedance buffer-based reactance cancellation method for clc-s compensated wireless power transfer. IEEE Trans Industr Electron. 2024;71(7):6894–906. 10.1109/TIE.2023.3314863.

[64] Cho ZH, Yi JH. Planar surface gradient coil. Concepts Magnet Resonance. 1995;7:95–114. 10.1002/cmr.1820070202.

[65] Giovannetti G, Flori A, De Marchi D, Matarazzo G, Frijia F, Burchielli S, et al. Simulation, design, and test of an elliptical surface coil for magnetic resonance imaging and spectroscopy. Conc Magn Resonance Part B. 2017;47B:21361. 10.1002/cmr.b.21361.

[66] Xiao-Liang Y, Zhong L, Min L, Guang-Yao L, Qiang K, Jie T, et al. Design of sc solenoid with high homogeneity. Chin Phys C. 2014;38(3):037001. 10.1088/1674-1137/38/3/037001.

[67] Awuah CM, Danuor P, Moon JI, Jung YB. Novel coil design and analysis for high-power wireless power transfer with enhanced q-factor. Sci Rep. 2023;13:4187. 10.1038/s41598-023-31389-y. PMC10014958 36918669

[68] Mollaei MSM, Van Leeuwen CC, Raaijmakers AJE, Simovski CR. Analysis of high impedance coils both in transmission and reception regimes. IEEE Access. 2020;8:129754–62. 10.1109/ACCESS.2020.3009367.

[69] Zinn S, Semiatin SL. Elements of induction heating: design, control, and applications. 1st ed. Materials Park: ASM International; 1988.

[70] Cui Y, Wang Y, Huang Q, Wei S. Effect of radiation and convection heat transfer on cooling performance of radiative panel. Renewable Energy. 2016;99:10–7. 10.1016/j.renene.2016.06.025.

[71] Lupi S, Forzan M, Aliferov A. Induction and direct resistance heating: theory and numerical modeling. 1st ed. Cham: Springer; 2015.

[72] Optris. https://optris.com/. Accessed on 09 May 2026.

[73] Chakraborty D, Brezovich I. Error sources affecting thermocouple thermometry in rf electromagnetic fields. J Microwave Power. 1982;17(1):17–28. 10.1080/16070658.1982.11689261. 6921255

[74] Yusuf GT, Fang C, Huang DY, Sellars ME, Deganello A, Sidhu PS. Endocavitary contrast enhanced ultrasound (ceus): a novel problem solving technique. Insights Imag. 2018;9(1):303–11. 10.1007/s13244-018-0601-x. PMC5991005 29594851

[75] Verasonics. https://verasonics.com/. Accessed on 09 May 2026.

[76] Skumiel A, Leszczynski B, Molcan M, Timko M. The comparison of magnetic circuits used in magnetic hyperthermia. J Magn Magn Mater. 2016;420:177–84. 10.1016/j.jmmm.2016.07.018.

[77] Vicentini M, Ferrero R, Manzin A. Influence of coil geometry, supply conditions and nanoparticle heating properties on magnetic hyperthermia in mouse models. Int J Therm Sci. 2024;203:109151. 10.1016/j.ijthermalsci.2024.109151.

[78] Gupta A, Yadav AK. Numerical analysis of solenoidal and helmholtz coils for magnetic hyperthermia with intratumoral nanofluid injection. ASME J Med Diagn. 2026;9(4):041201. 10.1115/1.4071394.

[79] Brizi D, Fontana N, Giovannetti G, Menichetti L, Cappiello L, Doumett S. A radiating system for low-frequency highly focused hyperthermia with magnetic nanoparticles. IEEE J Electromagn RF Microwav Med Biol. 2020;4(2):109–16. 10.1109/JERM.2019.2945833.

[80] Skumiel A, Wojciechowski RM. Two-phase system for generating a higher-frequency rotating magnetic field excited causing hyperthermic effect in magnetic fluids. Energies. 2022;15(22):8326. 10.3390/en15228326.

[81] Wojciechowski RM, Skumiel A, Kurzawa M, Demenko A. Design, application and investigation of the system for generation of fast changing, rotating magnetic field causing hyperthermic effect in magnetic liquids. Measurement. 2022;194(22):111020. 10.1016/j.measurement.2022.111020.

[82] Skumiel A. Comparison of two- and three-phase devices generating a rotating magnetic field for magnetic hyperthermia applications. Magnetism. 2025;5(3):21. 10.3390/magnetism5030021.

[83] Iszály Z, Márián IG, Szabó IA, Trombettoni A, Nándori I. Theory of superlocalized magnetic nanoparticle hyperthermia: rotating versus oscillating fields. J Magn Magn Mater. 2022;541:168528. 10.1016/j.jmmm.2021.168528.

[84] Giustini AJ, Petryk AA, Cassim SM, Tate JA, Baker I, Hoopes PJ. Magnetic nanoparticle hyperthermia in cancer treatment. Nano LIFE. 2010;1(01n02):17–32. 10.1142/S1793984410000067. PMC3859910 24348868

[85] Chang D, Lim M, Goos JACM, Qiao R, Ng YY, Mansfeld FM, et al. Biologically targeted magnetic hyperthermia: potential and limitations. Front Pharmacol. 2018;9:831. 10.3389/fphar.2018.00831. PMC6083434 30116191

[86] Pieterman K, Dam JAM, Postma H. Low temperature closed cooling system for MRI magnets. In: Fast RW, editor. Adv Cryog Eng, vol. 31. Boston, MA: Springer; 1986. 10.1007/978-1-4613-2213-9_61.

[87] Kim ES, Park SM. Enhanced modeling of load behavior in high-efficiency dual-coil induction heating systems. J Power Electr. 2025;25:2144–52. 10.1007/s43236-025-01177-0.

[88] Alamouti AK, Habibi MR, Sharfabadi MM, Lalimi HA. Numerical study on the effects of blood perfusion and tumor metabolism on tumor temperature for targeted hyperthermia considering a realistic geometrical model of head layers using the finite element method. SN Appl Sci. 2021. 10.1007/s42452-021-04447-1.

[89] Sadeghi F, Zarandi HRM, Ahangar HA, Mollaei MH, Torabi M, Ghasemi M, et al. A comprehensive modeling on thermal damage in tumor hyperthermia therapies using magneto-plasmonic nanocomposite. Sci Rep. 2025. 10.1016/j.phymed.2020.153402. PMC12264184 40665011

[90] Bosque JJ, Calvo GF, Perez-Garcia VM, Navarro MC. The interplay of blood flow and temperature in regional hyperthermia: a mathematical approach. Roy Soc Open Sci. 2021;8(1):201234. 10.1098/rsos.201234. PMC7890498 33614070

[91] Stigliano RV, Shubitidze F, Petryk JD, Shoshiashvili L, Petryk AA, Hoopes PJ. Mitigation of eddy current heating during magnetic nanoparticle hyperthermia therapy. Int J Hyperth. 2016;32(7):735–48. 10.1080/02656736.2016.1195018. PMC5095930 27436449

[92] Kwok MKY, Maley CCJ, Dworkin A, Hattersley S, Southern P, Pankhurst QA. Nonspecific eddy current heating in magnetic field hyperthermia. Appl Phys Lett. 2023;122(24):240502. 10.1063/5.0153336.

[93] Wang C, Li S, Zeng Z, Wang A, Wang S, Li C, et al. A triple-axis hybrid-linearity selective gradient field system design in magnetic hyperthermia: providing a spheroidal shaped field-free region. J Appl Phys. 2026;139:085101. 10.1063/5.0306597.

[94] Carlton H, Weber M, Peters M, Arepally N, Lad YS, Jaswal A, et al. Hyper: pre-clinical device for spatially-confined magnetic particle hyperthermia. Int J Hyperth. 2023;40(1):085101. 10.1080/02656736.2023.2272067. PMC10624165 37875265

